# Electrochemical Determination of Chloroquine Phosphate in Real Samples Using a Diresorcinate‐1,10‐phenanthrolinecobalt(II)‐Modified Glassy Carbon Electrode

**DOI:** 10.1002/open.202300004

**Published:** 2023-03-27

**Authors:** Adane Kassa, Getinet Tamiru Tigineh, Atakilt Abebe

**Affiliations:** ^1^ Department of Chemistry College of Natural and Computational Sciences Debre Markos University 269 Debre Markos Ethiopia; ^2^ Department of Chemistry College of Science Bahir Dar University 79 Bahir Dar Ethiopia

**Keywords:** chloroquine phosphate, diresorcinate-1,10-phenanthrolinecobalt(II) complex, electropolymerization, sensor, voltametric determination

## Abstract

Chloroquine phosphate (CQP) is used for malaria treatment. As it is facing increasing resistance, it needs continuous monitoring using sensitive and specific detection methods. In this work, a voltammetric sensor was prepared by electropolymerization of a diresorcinate‐1,10‐phenanthrolinecobalt(II) complex on a glassy carbon electrode (poly(DHRPCo)/GCE) which was followingly characterized. Compared with a bare GCE, CQP showed single well shaped irreversible oxidative peak at the poly(DHRPCo)/GCE. The peak current showed excellent linearity with CQP concentration in the range of 0.005–300.0 μm with a detection limit of 0.39 nm. The response of CQP at poly(DHRPCo)/GCE was not influenced by the presence of amoxicillin, ciprofloxacillin and paracetamol in addition to its high stability and reproducibility. It was applied for detection of CQP in various real samples, including three brands of tablets, human blood serum, and urine samples. The detected amount in tablets were in the range 98.4–103.2 % of their labeled value. Spike recovery results in human blood serum, urine, and tablet samples were 99.35–100.28 %, 99.03–100.32 %, and 98.40–100.41 %, respectively. Interference recovery results with less than 4.60 % error, the lower limit of detection and the wider dynamic range than most of the previously reported methods validate the potential applicability of the proposed method for CQP determination in various real samples with complex matrices.

## Graphical abstract

**The synthesis, characterization and electrochemical application** of a novel mixed ligand Co(II) complex for SWV determination of Chloroquine phosphate CQP in biological and pharmaceutical formulations samples are reported. The developed sensor is validated by recovery, interference, and long‐term stability and reproducibility tests. The method showed excellent performance, making it an excellent candidate for determination of CQP in real samples.

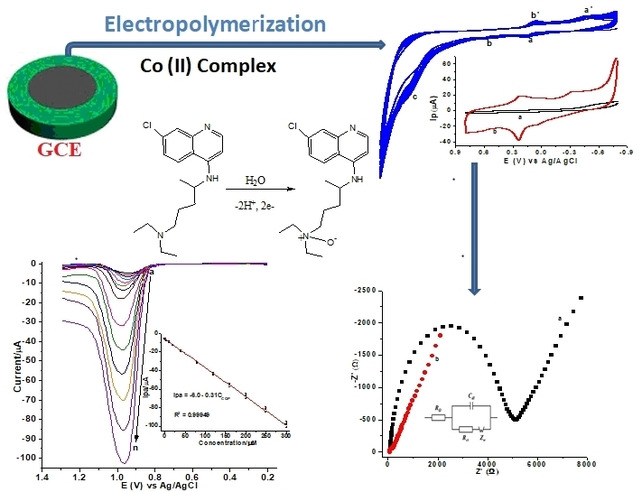

## Introduction

**Scheme 1:**
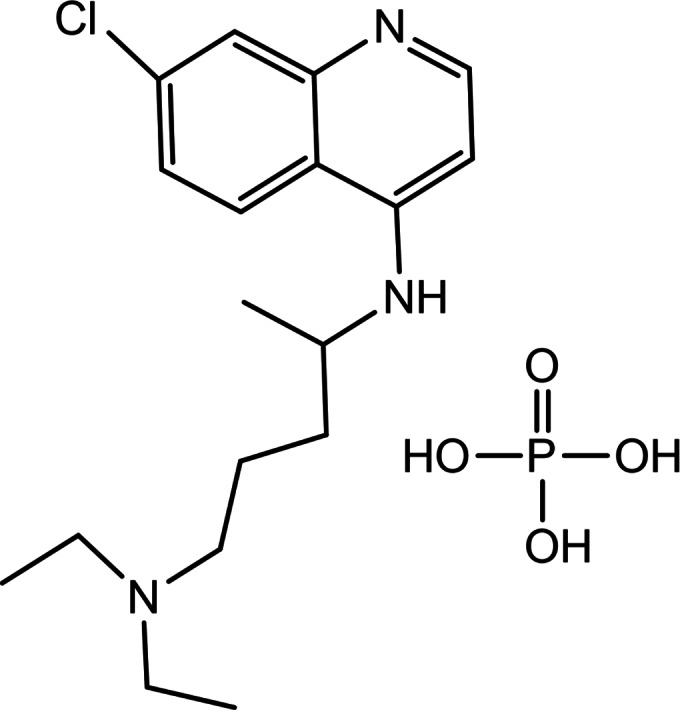
Structure of CQP.

Chloroquine phosphate (7‐chloro‐4‐4‐diethylamino‐1‐methylbutyl amino‐quinoline diphosphate) (Scheme 1), is the derivative of quinolines,^[1]^ which was used for the treatment of including malaria, amebiasis, polymyositis, and photosensitive diseases.[^2^, ^3^, ^4^, ^5^] It has been suggested as a potential anticancer drug,^[6]^ and for treatment of COVID‐19.[^1^, ^7^, ^8^] Several studies suggested that interactions of chloroquine with DNA might underlie the antimalarial activity of CQP.^[6]^

Side effects of CQP include cardiac arrest, kidney failure, blindness, heart arrhythmias, and cerebral edema, ophthalmic complications and sudden death.[^9^, ^10^] These and other disorder may arise due to miss‐use such as high drug dosage, over prescription and poor patient compliance. CQP has wide clinical potential that preparing sensitive and selective detection methods for monitoring CQP levels in tablet and biological samples such as blood serum and urine is important. This is helpful to study its pharmacokinetics. This is important to reach at decisions whether malaria developed resistance to this medication and alternative solutions.[^4^, ^7^, ^8^]

Different analytical methods including spectrophotometry,[^11^, ^12^] spectrofluorometry,^[5]^ LC–MS/MS,^[2]^ HPLC‐DAD,^[3]^ HPLC,^[13]^ GC‐MS,^[14]^ and capillary electrophoresis^[15]^ are reported for the quantification of CQP in biological fluid samples and pharmaceutical forms. However, these methods show some disadvantages such as extended analysis time, complicated sample preparation steps, required trained manpower, high instrumental and analysis cost, which also contributes to the more significant production of chemical residues.[^17^, ^18^] Yet, electrochemical methods can be easily operated, requiring lower‐cost equipment, showing high sensitivity and precision, environmentally friendly and quick response.[^16^, ^17^, ^18^, ^19^, ^20^, ^21^]

Electrochemical sensors can be manufactured by using several materials, and in some cases, taking many steps through the synthesis of modifiers.[^20^, ^22^] Recently, transition metal complex electro polymerization of on GCE has drawn a lot of interest from researchers due to its simple electrode modification process, cheap, and low toxicity. The use of transition metal coordination compounds as electrode modifiers attributed to electrocatalytic activity, electrical conductivity, reproducibility, improved sensitivity, larger surface area, and strong interaction with analyte than the bare electrode, making them excellent candidates to act as electrode modifiers.[^16^, ^18^]

Limited studies on the electrochemical determination of CQP in pharmaceutical and biological fluid samples have been reported using modified electrodes such as DNA modified carbon paste electrode,^[6]^ boron‐doped diamond (BDD),^[17]^ reduced graphene oxide on WS_2_ quantum dots (rGO@WS_2_/GCE),^[20]^ a multi‐walled carbon nanotube modified carbon paste (MWCNTs/CPE),^[9]^ carbon‐clay paste doped with Titanium oxide (CPEA/TiO_2_),^[23]^ and polymer modified GCE are obtained.^[24]^ However, these sensors need expensive materials and complex preparation procedures. Thus, developing a simple and sensitive method for accurate quantification of CQP in various real samples is necessary.

Hence, in this work, a sensitive stripping electrochemical method was employed for the CQP determination in tablets and biological samples with GCE modified with diresorcinate‐1,10‐phenanthrolinecobalt(II) complex, which was synthesized using non‐toxic reagents (resorcinol, 1,10‐phenanthroline, and CoCl_2_) and requires one step for its production in comparison to other electrodes. By monitoring the non‐formation of white precipitate when the aqueous solution of the complex was treated with an aqueous silver nitrate solution, it was possible to verify that the modifier complex did not include counter chloride.

## Materials and Methods

### Chemicals and apparatus

Chloroquine phosphate (≥99 %, Sigma‐Aldrich), 1,10‐phenanthroline monohydrate (≥99.7 %, Sigma Aldrich), silver nitrate (≥99.0 %, Sigma‐Aldrich), potassium hexacyanoferrate(II) and potassium hexacyanoferrate(III) (98.0 %, BDH laboratories supplies, England), resorcinol (≥99.0 %, BDH laboratories supplies, England), potassium chloride (99.5 %, Blulux laboratories (p) Ltd), sodium monohydrogen phosphate and sodium dihydrogen phosphate (≥98 %, Blulux laboratories (p) Ltd), sulfuric acid (98 % Loba Chemie, laboratory reagent), hydrochloric acid (37 %, Fisher Scientific), and sodium hydroxide (Extra pure, Lab Tech Chemicals) were used without further purification.

CH Instruments 760E potentiostat (Austin, Texas, USA), pH meter (AD8000, Adwa, Romania), deionizer (Evoqua water technologies, Germany), centrifuge (1020D, Centurion scientific LTD, UK), electronic balance (Nimbus, ADAM equipment, USA) were among the instruments used.

### Electrochemical Procedures

Electrochemical measurements were performed using a conventional three electrode system whereby poly(DHRPCo)/GCE or unmodified GCE as working electrode, Ag/AgCl (3.0 m KCl) as reference electrode and Pt coil as counter electrode. Prior to every measurement, the working electrode was regenerated by scanning in blank solution until stable voltammogram was obtained. While electrochemical impedance spectroscopy (EIS) and cyclic voltammetry (CV) were used to characterize the modified electrode and/or investigate the electrochemical behavior of CQP at poly(DHRPCo), AdSSWV was employed for quantitative determination of CQP in the analyzed human blood serum, human urine, and three brands of tablet formulation samples.

### Electrochemical preparation of poly(DHRPCo)/GCE

Poly(DHRPCo)/GCE was prepared following the reported electropolymerization procedure with substantial modification.^[18]^ The GCE was polished to a mirror finish with alumina slurry and gently washed with deionized water. Electropolymerization of DHRPCo on the surface of the polished GCE was accomplished in 1.0 mm DHRPCo in 0.1 m PBS of pH 7.0 between −0.8 and +1.8 V for 20 cycles at a scan rate of 100 mV s^−1^. The resulting modified electrode was rinsed with distilled water and scanned in monomer‐free 0.5 m H_2_SO_4_ between −0.8 and +0.8 V until a steady‐state CV was obtained.

### Preparation of standard solutions

A stock solution (5.0 mm) of CQP was prepared by dissolving 257.95 mg of CQP in deionized water 100 mL of a volumetric flask. The working solution was prepared from the stock solution through serial dilution with 0.1 m PBS of the appropriate pH.

### Preparation of real samples

Three commercially available CQP tablets were purchased from local drug stores in Bahir Dar city, Ethiopia. Five weighed CQP tablets from each of the three tablet brands including; Addis pharmaceutical factory (APF), Ethiopia; Bayer Zydus Pharma Pvt Ltd (Resochin), India; and IPCA Laboratories Ltd (Lariago), India, with an average tablet mass of 307.60, 315.16, and 297.54 mg/tablet, respectively were ground and homogenized using a mortar and pestle. Stock solutions (2.0 mm) of the tablet were prepared by dissolving (126.95, 130.07, and 122.80 mg of APF, Resochin, and Lariago, respectively) tablet powder into a 100 mL volumetric flask and filled up to the mark with deionized water. Working solutions of CQP were further prepared from stock solution of each brand in pH 7.5 PBS.

The human blood serum and urine samples were obtained ready‐to‐use from Tibebe Gion referral hospital at Bahir Dar City, which follows best practices in volunteer sample collection. The samples were transferred to 25 mL conical flask, filled to the mark with pH 7.5 PBS and stored in refrigeration for further analysis.

## Results and Discussion

### Synthesis of poly(DHRPCo)/GCE

#### Optimization of potential window

**Figure 1:**
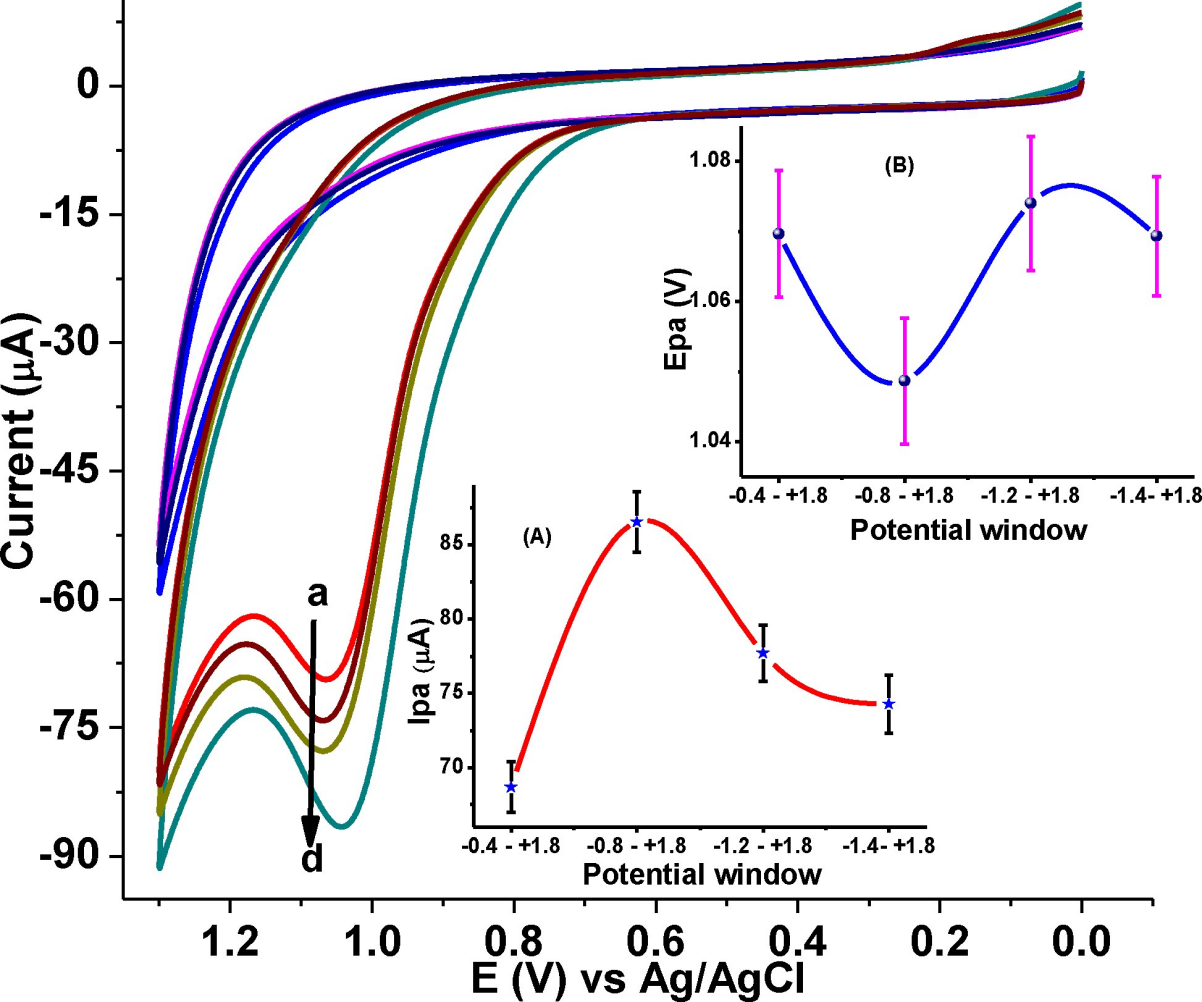
CVs of pH 7.0 PBS containing 1.0 mm CQP (a‐d) at poly(DHRPCo)/GCE synthesized by scanning the potential between (a) −0.4&+1.8 V, (b) −1.4&+1.8 V, (c) −1.2&+1.8 V, and (d) −0.8&+1.8 V at 100 mV s^−1^ for 15 cycles. Inset: plot of peak current (A), and peak potential (B) of CQP vs. potential window range.

The electropolymerization of 1.0 mm DHRPCo in PBS of pH 7.0 on the GCE was carried out at potentiodynamically various potential windows (−0.4 to +1.8 V, −0.8 to +1.8 V–1.2 to +1.8 V and −1.4 to +1.8 V) for 15 cycles. From the Figure 1, the highest oxidative peak current of 1.0 mm CQP was recorded within the potential range of −0.8 to +1.8 V. Hence, due to current and over‐potential advantages, the optimized potential window for the electropolymerization of DHRPCo at GCE was between −0.8 to +1.8 V.

#### Optimization of film thickness

**Figure 2:**
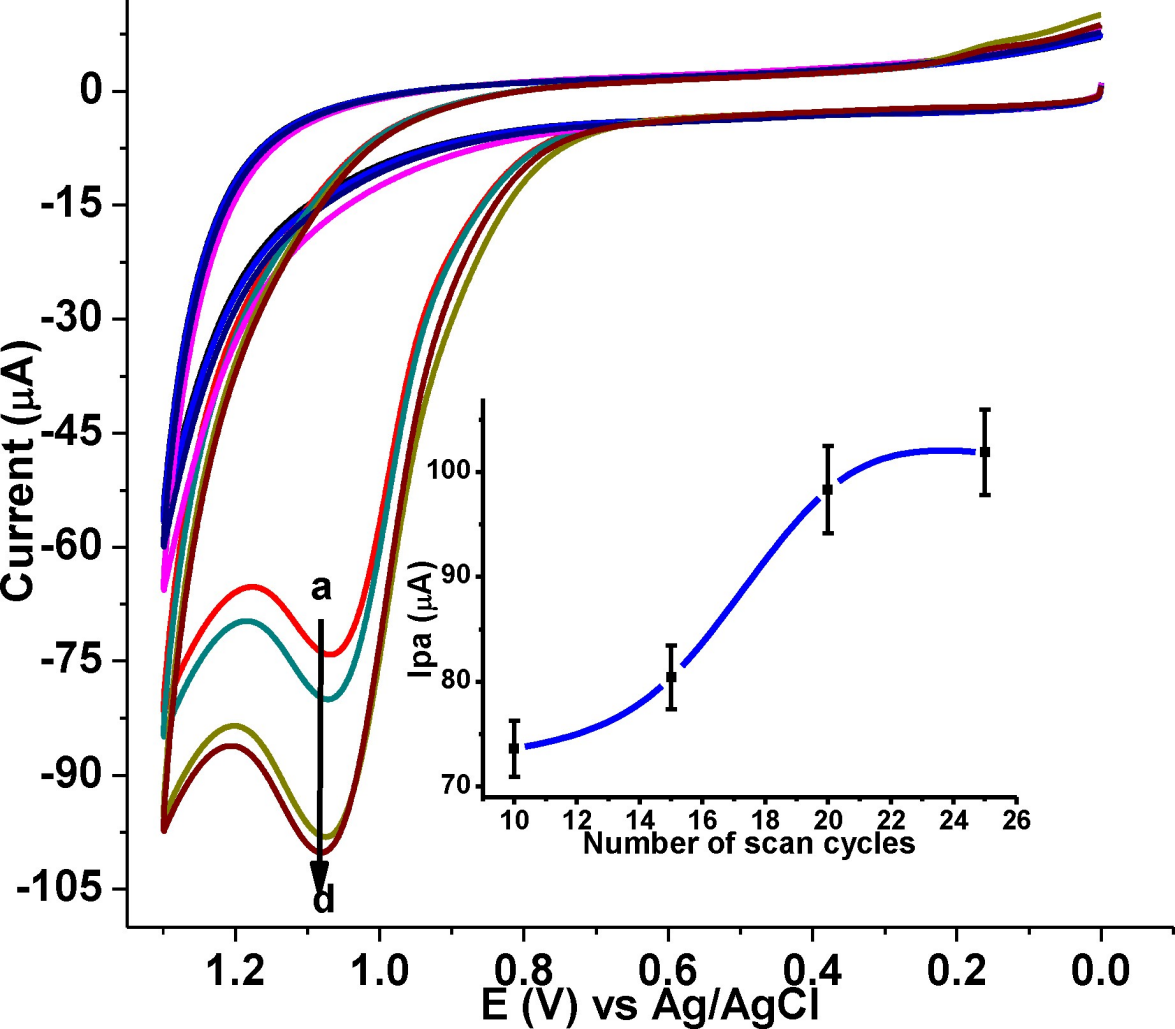
CVs of 1.0 mm CQP in pH 7.0 PBS at poly(DHRPCo)/GCE for different number of polymerization cycles (a–d: 10, 15, 20, and 25, respectively). Inset: plot of I_pa_ vs. number of scan cycles.

The film thickness of the modified electrode was one of the most important parameters which affect the peak current of analytes. The oxidative peaks current of 1.0 mm CQP was increased with the increasing number of cycles, which may be due to the increase in effective surface area of GCE as a result of modification (Figure 2).[^16^, ^18^, ^21^] Even though a considerable enhancement of oxidative peaks current was observed at each cycle, largest increment with highest slope was recorded by 20 cycles (Inset of Figure 2), beyond 20 cycles slight increase of peak current suggests that the overdose of the complex film at the GCE reduces its interaction with the electroactive functional group of CQP due to the saturation of the active sites. Therefore, 20 cycles were selected as the optimal condition for preparation of poly(DHRPCo)/GCE for determination of CQP in human blood serum, human urine and pharmaceutical formulation samples.

**Figure 3:**
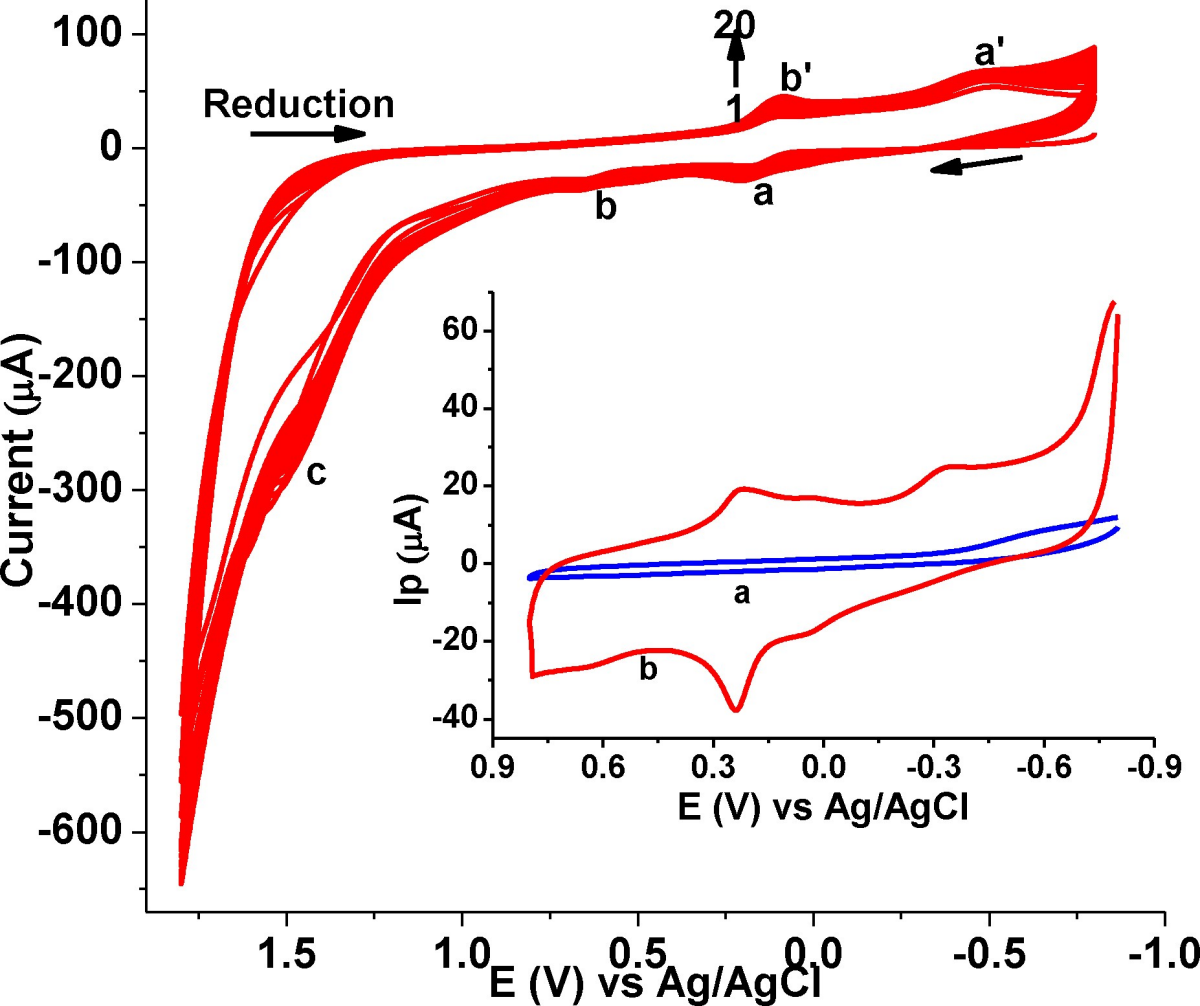
CVs of GCE in PBS of pH 7.0 containing 1.0 mm DHRPCo scanned between −0.8 V to +1.8 V at 100 mV s^−1^ for 20 cycles. Inset: CVs of (a) bare GCE and (b) poly(DHRPCo)/GCE in a 0.5 m H_2_SO_4_ scanned between −0.8 and +0.8 V.

Figure 3 shows the cyclic voltammograms of 1.0 mm DHRPCo in PBS pH 7.0 for 20 scan cycles at the GCE. In the scan, the anodic and cathodic peaks were observed which might correspond to the redox property of DHRPCo monomer at the surface of GCE.^[18]^

Increasing of peaks current in the successive scanning indicates the growth of electroactive polymeric film at the electrode surface. As shown clearly in the figure, the voltammogram of poly(DHRPCo)/GCE displayed distinct redox peaks (Inset b of Figure 3) which are not exhibited at the GCE, showing the immobilization of electroactive poly(DHRPCo) film at the surface of GCE.

### Characterization of the poly(DHRPCo)/GCE

#### Cyclic voltammetry characterization of poly(DHRPCo)/GCE

**Figure 4:**
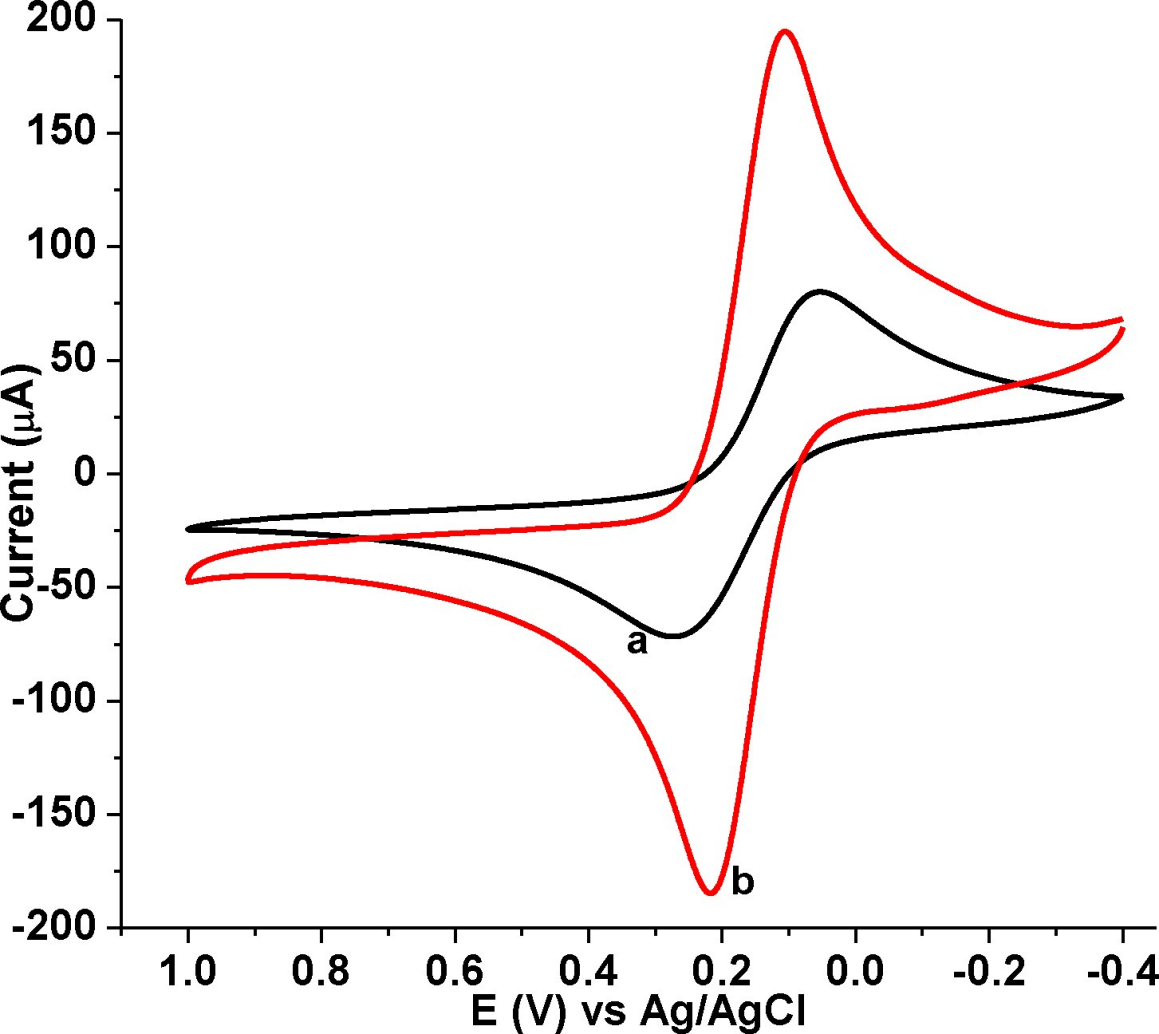
CVs of 10.0 mm [Fe(CN)_6_]^3−/4−^ in 0.1 m KCl (a) bare GCE, and (b) poly(DHRPCo)/GCE at scan rate of 80 mV s^−1^.

The electrochemical behavior of the modified electrode was examined in 10.0 mm [Fe(CN)_6_]^3−/4−^ solution in 0.1 m KCl using CV. As can be seen in Figure 4, both anodic and cathodic peak currents of [Fe(CN)_6_]^3−/4−^ at poly(DHRPCo)/GCE (curve b) were about three times higher than the currents recorded at the unmodified GCE (curve a). Besides, the peak‐to‐peak separations of GCE (ΔE=228.9 mV) and poly(DHRPCo)/GCE (ΔE=104.5 mV). This could be attributed to faster electron transfer kinetics and high electroactive surface area of the modified electrode compared with bare GCE.^[18]^

**Figure 5:**
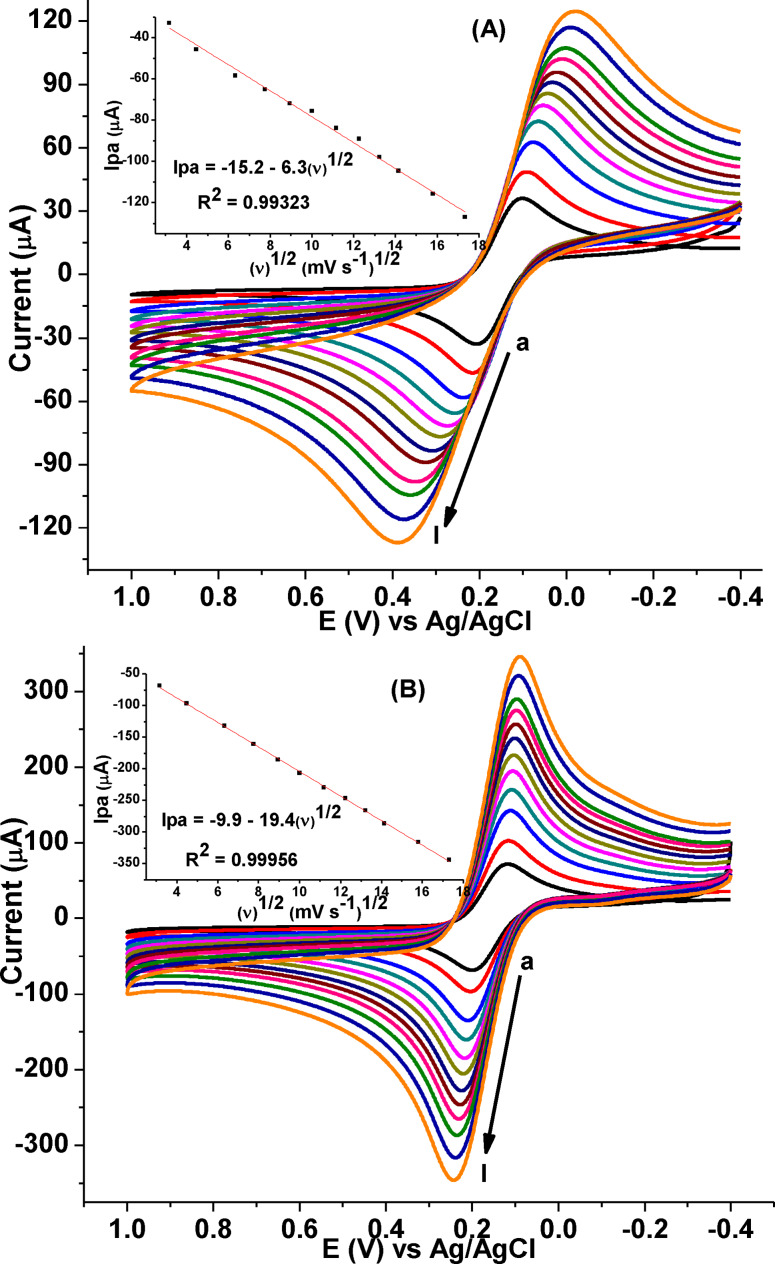
CVs of 10.0 mm [Fe(CN)_6_]^3−/4−^ in 0.1 m KCl (A) unmodified GCE, (B) poly(DHRPCo)/GCE at different scan rate (a‐i: 10, 20, 40, 60, 80,100, 125, 150, 175, 200, 250 and 300 mV s^−1^, respectively). Inset: plot of I_pa_ vs. (ν)^1/2^.

The electroactive surface area of the electrodes was calculated from the slope value of the plot of current versus square root of scan rate of Figure 5, according to Randles–Ševčík equation [Eq. 1].^[25]^

(1) $$I_{pa}=2.69\;x\;10^{5}n^{3/2}AD^{1/2}\nu^{1/2}C_{o}$$

Where n is the number of electrons transferred for [Fe(CN)_6_]^3−/4−^ (n=1), I_p_ is the peak current, A is the effective surface area, D is the diffusion coefficient of [Fe(CN)_6_]^3−/4−^ (D=7.6×10^−6^ cm^2^ s^−1^), *v* is the scan rate, C is the concentration of the [Fe(CN)_6_]^3−/4−^ (10.0 mm).

The electroactive surface area of poly(DHRPCo)/GCE (Figure 5B) was 0.262 cm^2^, which is approximately 3.0 times higher than that of the unmodified GCE, surface area of 0.085 cm^2^ (Figure 5A). The significant enhancement in electroactive surface area of the modified electrode is the evidence for the formation of a larger number of available electrochemical active sites on the surface of poly(DHRPCo)/GCE which offers more electrocatalytic surface for the detection for CQP.

#### Electrochemical impedance spectroscopy characterization

**Figure 6:**
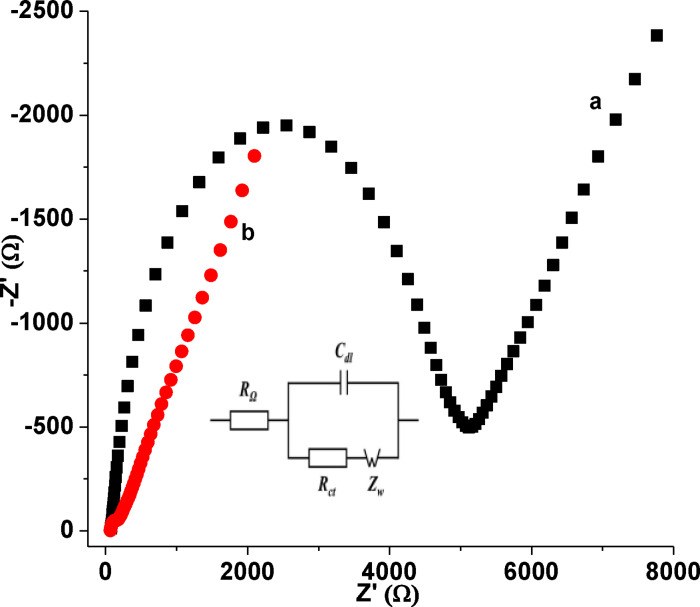
Nyquist plot of 10.0 mm [Fe(CN)_6_]^3−/4−^ in 0.1 m KCl at (a) GCE, and (b) poly(DHRPCo)/GCE in the frequency range: 0.01‐10,000 Hz, amplitude: 0.01 V and potential: 0.23 V. Inset: recommended equivalent circuit model.

Electrochemical impedance spectroscopy was used to investigate the interfacial properties of both poly(DHRPCo)/GCE and unmodified GCE.[^16^, ^18^] The EIS measurement was conducted in 10.0 mm [Fe(CN)_6_]^3−/4−^ in 0.1 m KCl over the frequency range of 0.01 to 10,000 Hz. As illustrated in Figure 6, the Nyquist plot of the GCE and poly(DHRPCo)/GCE consisted of semi‐circular and linear portions. The diameter of the semicircle at a higher frequency region represents the charge transfer resistance (R_ct_) of the electrode and the linear portion at the lower frequency region is an indicator of diffusion process of the electroactive species from the bulk to the solution‐electrode interface, all ruled by the recommended equivalent circuit (Inset of Figure 6).

The R_ct_ value of poly(DHRPCo)/GCE (206.6 Ω) was smaller than unmodified GCE (5427 Ω), indicating the surface of GCE greatly improved at poly(DHRPCo)/GCE and hence the electron transfer rate between the substrate and the analyte increase. The result obtained in EIS is in agreement with the results obtained in CV (Figure 5), which showed the successful deposition of poly(DHRPCo) at GCE surface.

Circuit elements including solution resistance (R_s_), R_ct_, and double layer capacitance (C_dl_) for each studied electrode as calculated from the respective Nyquist plot using Equation (2), and the apparent heterogeneous electron transfer rate constant (k^0^) calculated using eq. 3,[^16^, ^18^] are summarized (Table 1).

(2) $$C_{dl}=\;\frac{1}{{2\pi R}_{Ct}f}$$

(3) $$k^{0}=\;\frac{RT}{F^{2}{ACR}_{ct}}$$

**Table 1:** Summary of calculated circuit elements of EIS measurement.

Electrode	*R*_s_ [Ω cm^2^]	*R*_ct_ [Ω cm^2^]	Cdl [F]	f [Hz]	k^0^
GCE (curve a)	37.5	5427	9.28×10^−8^	316.2	5.8×10^−9^
poly(DHRPCo) (curve b)	37.5	206.6	1.37×10^−5^	56.2	4.9×10^−8^

Where C_dI_ – double layer capacitance, f – frequency corresponding to the maximum imaginary impedance (reactance) value on Nyquist plot, R_ct_ – charge transfer resistance, R – the molar gas constant (8.314 J mol^−1^ K^−1^), T – temperature (298 K), F – Faraday constant (96485 C mol^−1^), A – the surface area, and C – the concentration of [Fe(CN)_6_]^3−/4−^ (10.0 mm).

As can be seen from Table 1, 8.5 times higher k^0^ value for the poly(DHRPCo)/GCE than the unmodified GCE clearly indicates the electrocatalytic property of the modified electrode towards the probe.

### Cyclic voltammetric investigation of CQP

#### Electrochemical behavior of CQP at poly(DHRPCo)/GCE

**Figure 7:**
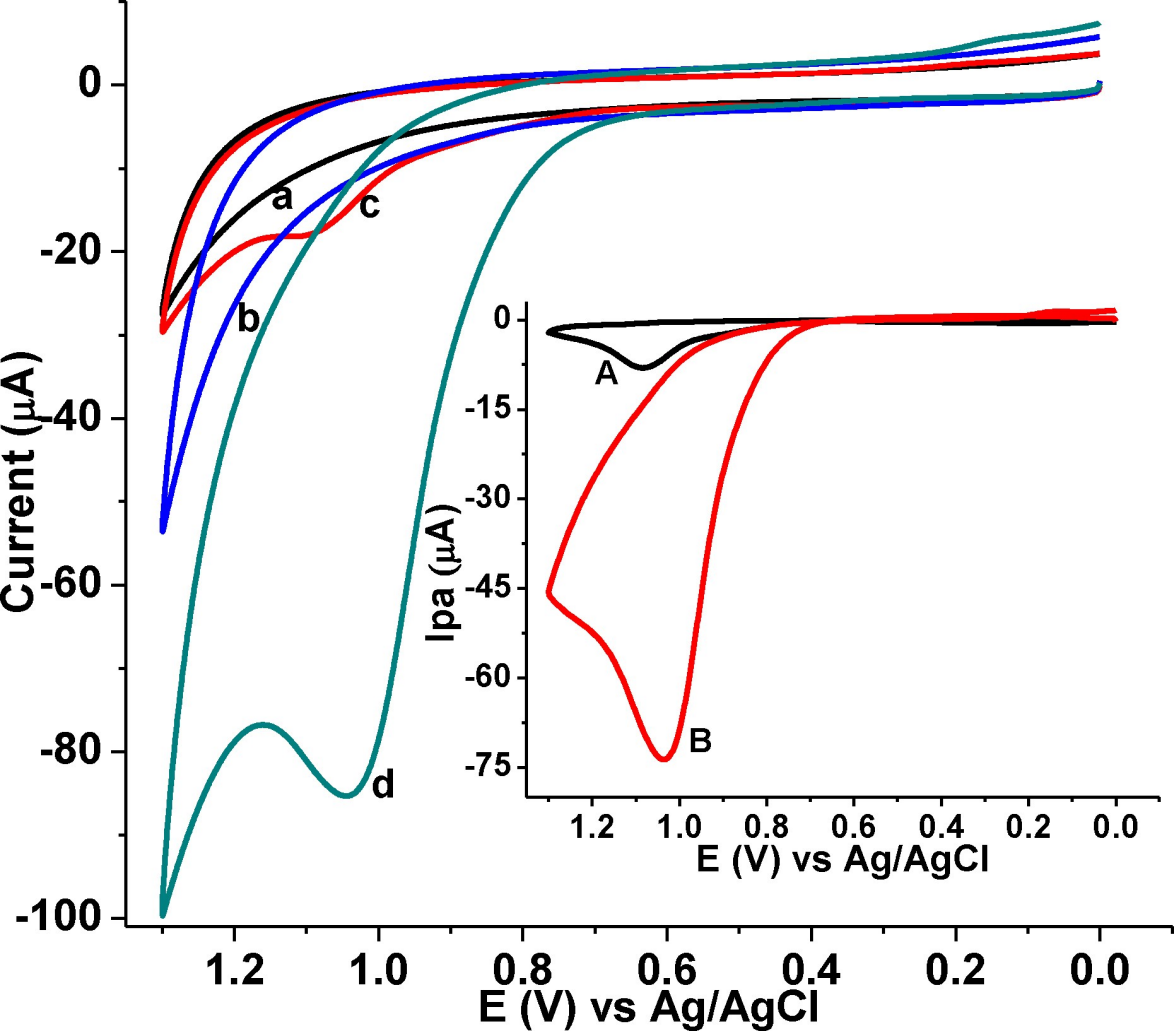
CVs of GCE (a & c) and poly(DHRPCo)/GCE (b & d) in the absence (a & b) and presence (c & d) of 1.0 mm CQP in pH 7.0 PBS at a scan rate of 100 mV s^−1^. Inset: blank subtracted CVs at GCE (a), and poly(DHRPCo)/GCE (b).

From the obtained characterization result (enhanced effective surface area, and low R_ct_) values of the poly(DHRPCo)/GCE confirmed excellent catalytic property of an electrode surface, its catalytic effect towards CQP was examined. The electrochemical behavior of 1.0 mm CQP in pH 7.0 PBS at GCE and poly(DHRPCo)/GCE was studied using CV (Figure 7).

However, CQP showed a highly enhanced well‐resolved single irreversible oxidation peak at much reduced potential with four folds current enhancement at the poly(DHRPCo)/GCE, indicated the catalytic effect of the poly(DHRPCo)/GCE towards oxidation of CQP. The absence of any reduction peak on the reverse scan indicated that the oxidation of CQP is irreversible at both the unmodified GCE and poly(DHRPCo)/GCE.

#### Effect of scan rate on E_pa_ and I_pa_ of CQP

**Figure 8:**
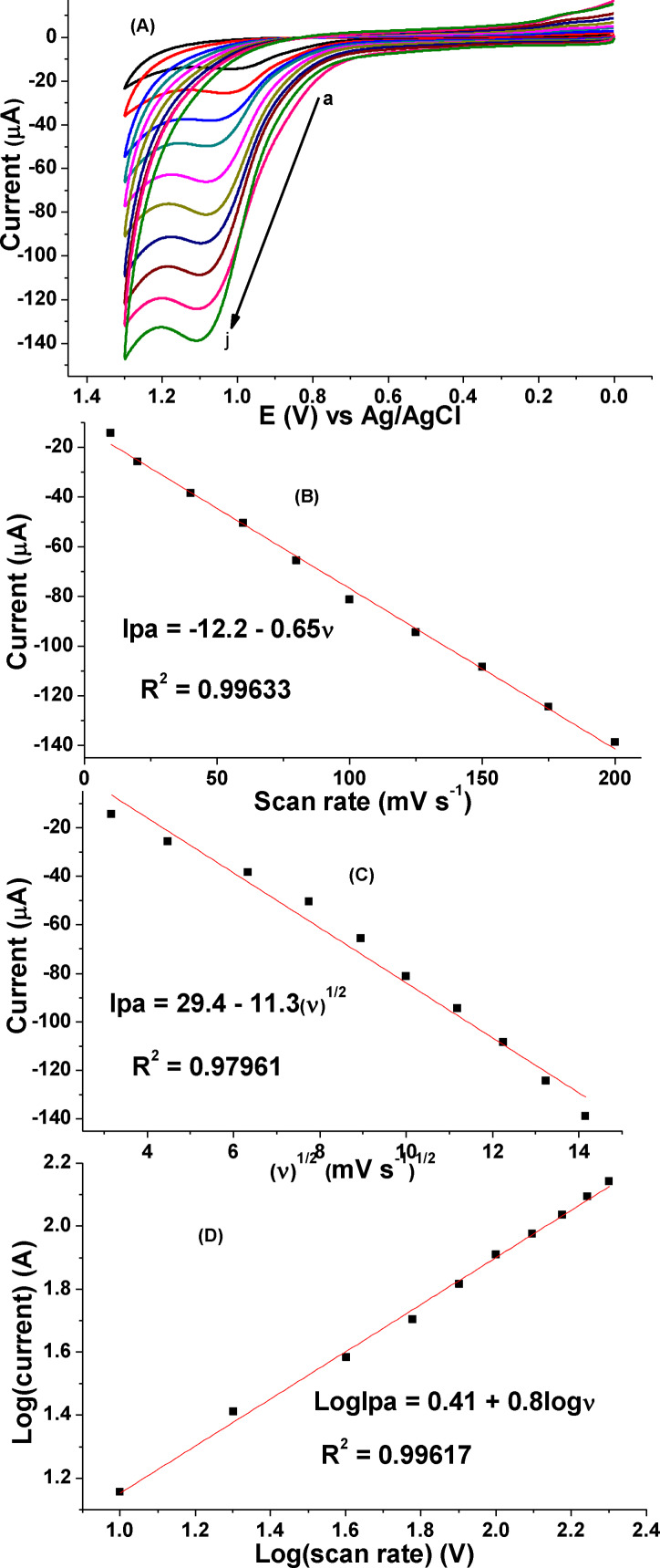
**(**A) CVs of 1.0 mm of CQP in PBS pH 7.0 at poly(DHRPCo)/GCE with various scan rates (a–j: 10, 20, 40, 60, 80, 100, 125, 150, 175, and 200 mV s^−1^, respectively), (B) plot of peak current vs. scan rate, (C) plot of peak current vs. square root of scan rate, and (D) plot of log(I_pa_) vs. log(ν).

The influence of scan rate on the peak potential and peak current of CQP in PBS of pH 7.0 solution was investigated using cyclic voltammograms in the range of 10–200 mV s^−1^ (Figure 8A). Higher correlation coefficient of the plot of oxidative peak current versus the scan rate (R^2^=0.99633) (Figure 8B) than with the square root of scan rate (R^2^=0.97961) (Figure 8C), suggested that oxidation of CQP at the poly(DHRPCo)/GCE was mainly controlled by the adsorption process.[^9^, ^17^] This was also confirmed by the slope of 0.8 for the plot of log peak current versus log scan rate (Figure 8D), which is exactly in agreement with the ideal value.^[26]^

While the observed peak potential shift of CQP with increasing the scan rate (Figure 8A) confirmed the irreversibility of the oxidation of CQP.^[19]^

The number of electron transferred during the electrochemical oxidation of CQP was calculated using Equation 4.^[25]^

(4) $$E_{p}-\;E_{\frac{p1}{2}}=\;\frac{48}{\alpha n}$$

The values of E_p_ and E_p1/2_ obtained from the cyclic voltammogram of CQP at 100 mV s^−1^ scan rate were 1029 and 974 mV E_p_ and E_p1/2_, respectively. The calculated value of *an* was 0.87. Since the value of *a* is 0.5 for a totally irreversible process,^[27]^ the calculated n value was 1.74, which is close to 2. This is in agreement with the reported literature.^[23]^

The charge transferred coefficient *(an)* of the electrode process during the electrochemical oxidation of CQP at poly(DHRPCo)/GCE was determined from the plot of E_pa_ versus ln(v) using Equation 5.^[25]^

(5) $$E_{p}=\;E^{0}+\;\frac{RT}{\left(1-\alpha \right)nF}\left\{0.780+\ln\left(\frac{D_{R}^{\frac{1}{2}}}{k^{0}}\right)+\;ln{\left[\frac{\left(1-\alpha \right)nF}{RT}\right]}^{\frac{1}{2}}\right\}$$

E_p_ ‐ oxidation peak potential, E^0^ – formal potential, α ‐ electron transfer coefficient, n ‐ number of electrons, k^0^ ‐ standard constant rate of the reaction, D_R_ – diffusion coefficient, ν – scan rate, T – temperature (298 K), R – molar gas constant (8.314 J mol^−1^ K^−1^), and F – Faraday constant (96,485 C mol^−1^).

**Figure 9:**
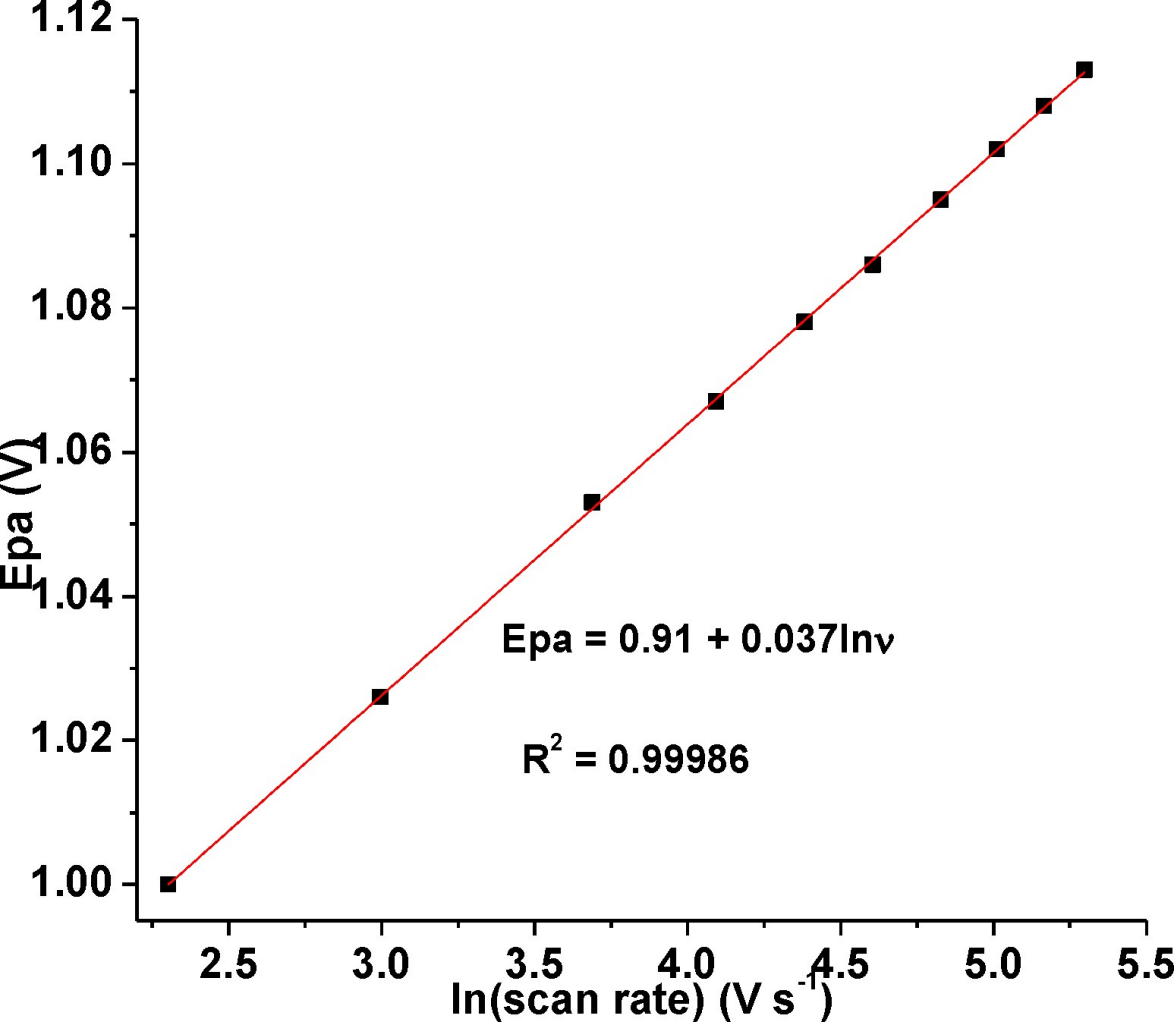
Plot of E_pa_ vs. ln(scan rate) containing 1.0 mm CQP in pH 7.0 PBS at poly(DHRPCo)/GCE.

From the slope of the plot of E_pa_ versus RT/2n(1‐α)Fln(ν) for the linear fit (Figure 9), the calculated value of n(1‐*a*) was 0.351. Taking the two electrons for oxidation of CQP calculated using Equation (5), the value of *a* was estimated to be 0.82, indicating the irreversibility of the oxidation of CQP at poly(DHRPCo)/GCE.^[18]^

#### Effect of pH on the oxidation of CQP

**Figure 10:**
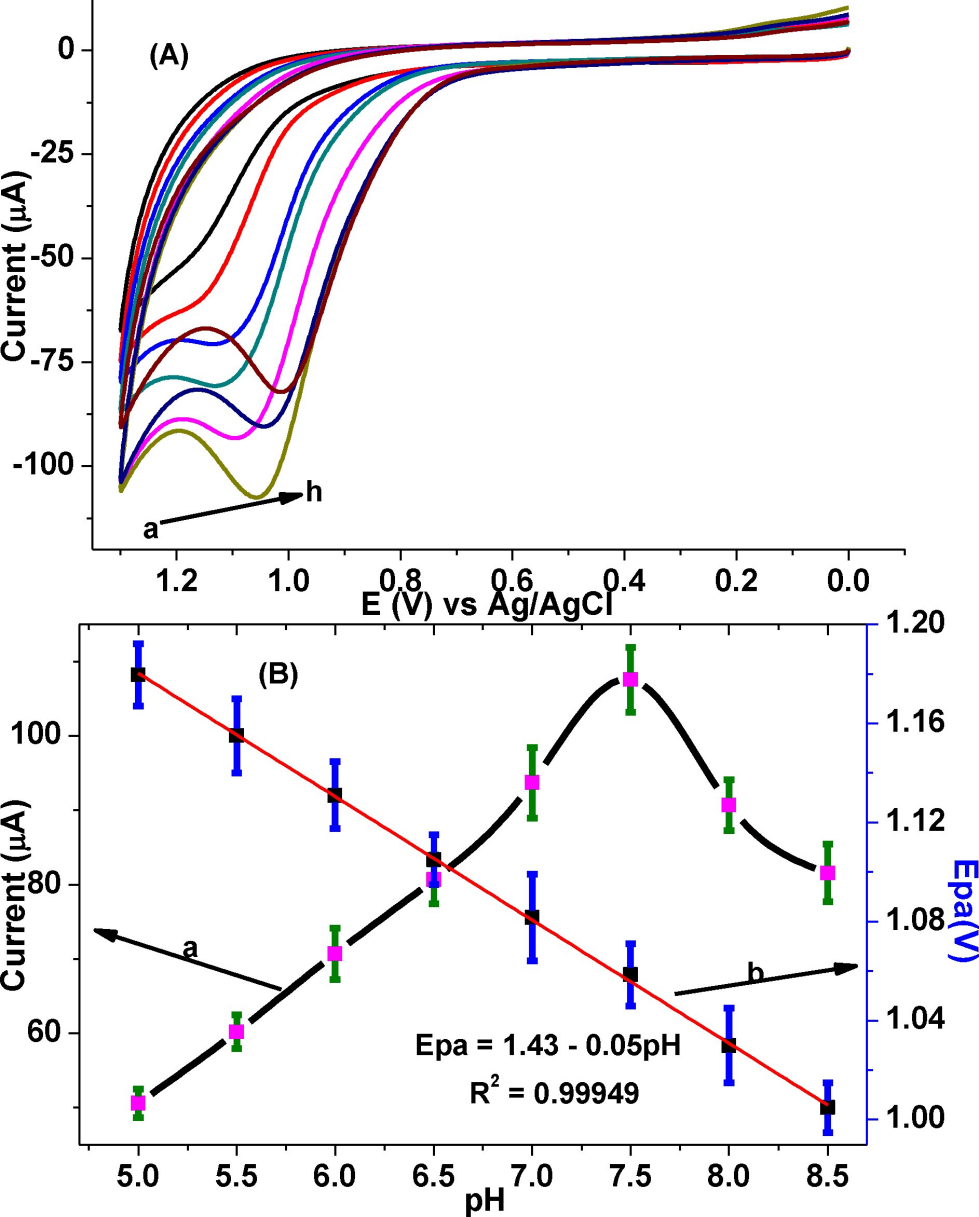
(A) CVs of poly(DHRPCo)/GCE containing 1.0 mm CQP in PBS of different pHs (a‐h: 5.0, 5.5, 6.0, 6.5, 7.0, 7.5, 8.0 & 8.5) at 100 mV s^−1^ scan rate, (B) plot of (a) I_pa_ and (b) E_pa_ vs. pH in the entire range.

The effect of pH on the peak current and peak potential of CQP was investigated to evaluate the participation of proton, proton:electron ratio, and rationalize the type of interaction between CQP and surface of the electrode (poly(DHRPCo)/GCE) in the pH range of 5.0–8.5 PBS (Figure 10A). The oxidation peak current of CQP at poly(DHRPCo)/GCE increased with pH from 5.0 to 7.5 and then decreased beyond pH values of 7.5 (curve b of Figure 10B), making pH 7.5 the optimum for further electrochemical investigations of CQP. The observed current increment at pH 7.5 could partly be attributed the strong electrostatic attraction of the cationic CQP (p*K*_a_: 8.4),^[1]^ with negatively charge poly(DHRPCo) film (phen, and reso with p*K*_a_ values of 4.86&9.15, respectively).[^16^, ^18^, ^21^]

The peak potential shift to the negative direction with pH values from 5.0 to 8.5 indicated proton participation in the oxidation process,^[28]^ the slope of peak potential versus pH of the PBS is 0.05 V (curve a of Figure 10B), which is very close to ideal Nernst value (0.059 V/pH), showed involvement of protons and electrons in 1 : 1 ratio during the reaction process.[^16^, ^18^, ^23^]

**Scheme 2:**
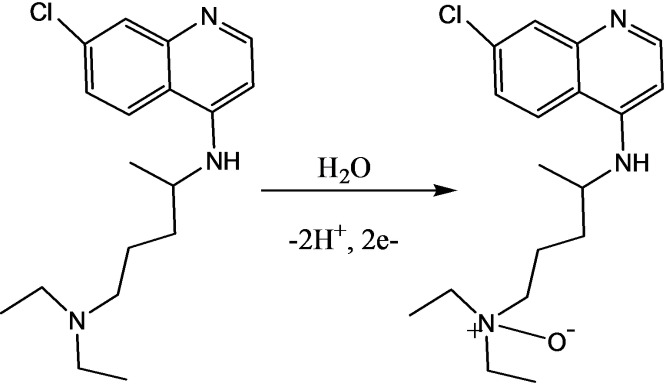
The proposed reaction mechanism of CQP oxidation.

Based on the calculated kinetic parameters (number of electrons and protons), and the electron to proton ratio, a reaction mechanism was proposed (Scheme 2), which is in agreement with the mechanism previously reported in literature,^[23]^ for the oxidation of CQP at poly(DHRPCo)/GCE.

### Adsorptive stripping square wave voltammetric determination of CQP at poly(DHRPCo)/GCE

#### Influence of accumulation potential and time

Since the electrochemical oxidation of CQP at poly(DHRPCo)/GCE is governed predominantly by adsorption controlled process, the effects of accumulation potential (E_acc_) and accumulation time (t_acc_) on the oxidation peak current for 1.0 mm CQP were investigated.

**Figure 11:**
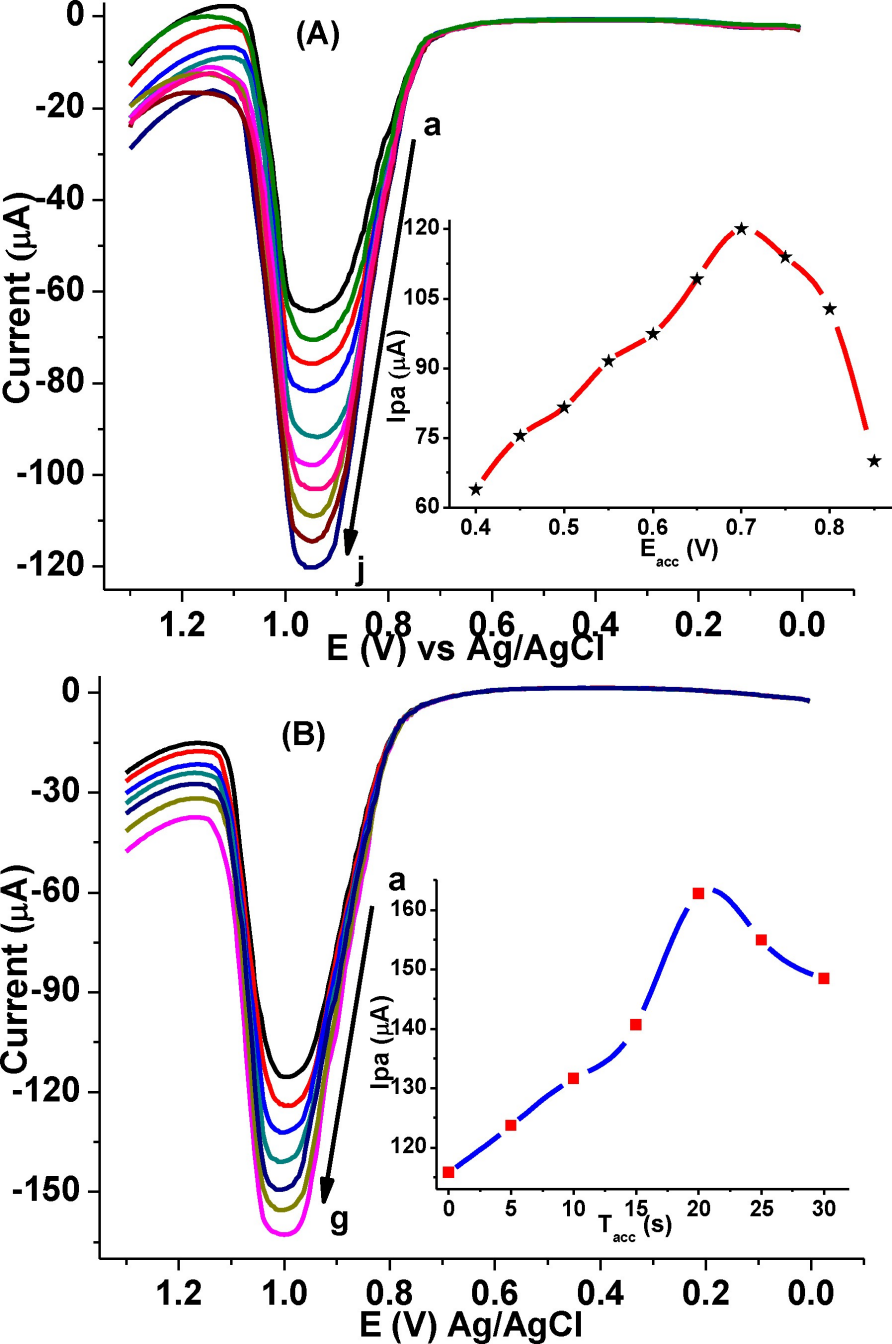
(A) Effect of E_acc_ at various potential (a‐k: 400, 450, 500, 550, 600, 650, 700, 750, 800, and 850 mV, respectively) at 10 s, and (B) Effect of t_acc_ at different times (a–f: 0, 5, 10, 15, 20, 25, and 30 s, respectively) at 700 mV on oxidative peak current of 1.0 mm CQP in pH 7.5 PBS at poly(DHRPCo)/GCE. Inset: plot of I_pa_ vs. E_acc_ (A), and t_acc_ (B).

Effect of E_acc_ at different deposition potentials in the potential range of 400 to 850 mV at a deposition time of 10 s (Figure 11A), the oxidation peak current of CQP initially increased when the accumulation potential was increased and reaches maximum at 700 mV further increase of the electrode potential a decrease in peak current is obtained.

On the other hand, evaluate the effect of accumulation time for the determination of CQP, variation of *t_acc_* between 0 to 30 s at an accumulation potential of 700 mV at pH of 7.5 (Figure 11B), the peak current increased with the increase in *t_acc_* up to 20 s, then decrease on any further increase of time. This suggests that the amount of adsorbed CQP on the modified electrode surface reaches a maximum value at 20 s (Inset of Figure 11B). Therefore, E_acc_ of 700 mV and *t_acc_* 20 s were chosen as the optimum accumulation potential and accumulation time for subsequent experiments.

**Figure 12:**
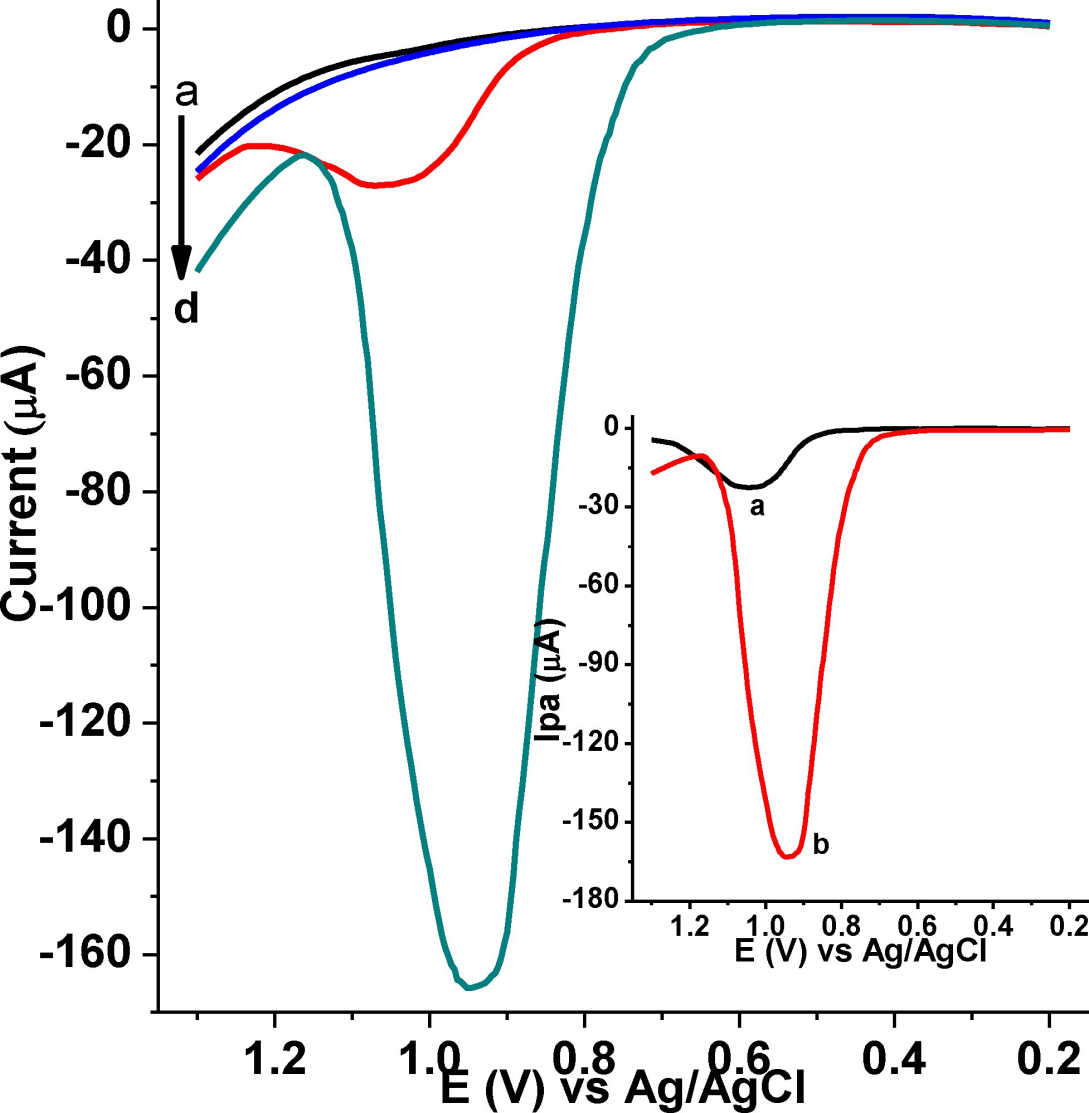
AdSSWVs of unmodified GCE (a & c) and poly(DHRPCo)/GCE (b & d) in pH 7.5 PBS containing no CQP (a & b) and 1.0 mm CQP (c & d) at E_acc_: 700 mV, t_acc_: 20 s, step potential: 4 mV, amplitude: 25 mV, and frequency: 15 Hz, Inset: blank corrected AdSSWVs of unmodified GCE (a), and poly(DHRPCo)/GCE (b).

Adsorptive stripping square wave voltammetric (AdSSWV) method, which is effective and rapid electroanalytical technique due to its ability to discriminate against back ground currents, good sensitivity and low detection limits,[^26^, ^29^, ^30^] was selected for the quantitative determination of CQP in tablet formulations, human blood serum, and urine samples. Figure 12 shows AdSSWV of 1.0 mm CQP in pH 7.5 PBS at bare GCE and poly(DHRPCo)/GCE. Appearance of a well‐shaped oxidative peak with much improved current at a reduced potential at poly(DHRPCo)/GCE (curve b of inset) than at the bare GCE (curve a of inset). These effects are a clear indication of the electro‐catalytic effect of the poly(DHRPCo) film for the oxidation of CQP.

#### Optimization of AdSSWV parameters

**Figure 13:**
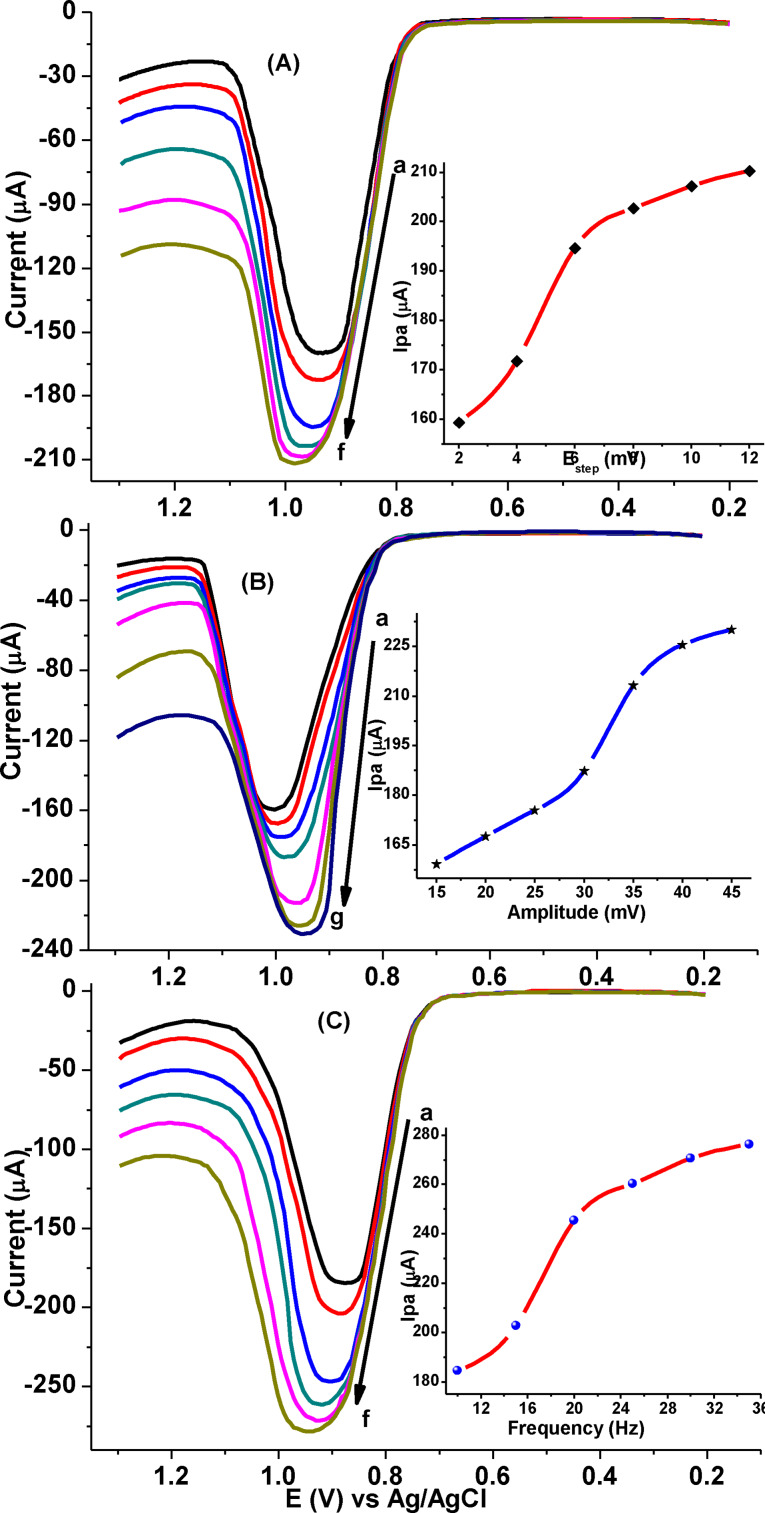
AdSSWVs of poly(DHRPCo)/GCE in pH 7.5 PBS containing 1.0 mm CQP at (A) various step potentials (a‐e: 2, 4, 6, 8, 10 and 12 mV, respectively), amplitude: 25 mV, and frequency:15 Hz, (B) various amplitudes (a‐g: 15, 20, 25, 30, 35, 40, and 45 mV, respectively), step potential: 6 mV, and frequency: 15 Hz, and (C) various frequencies (a‐f: 10, 15, 20, 25, 30 and 35 Hz, respectively), step potential: 6 mV and amplitude: 35 mV at E_acc_: 700 V, t_acc_: 20 s. Insert: plot of I_pa_ vs. step potential (A), amplitude (B), and frequency (C).

To develop the AdSSWV method for CQP analysis, square wave parameters (step potential, square wave amplitude, and frequency) were optimized. The effects of step potential, amplitude, and frequency on the peak current response were studied in the range from 2 to 12 mV, 15 to 45 mV and 10 to 35 Hz, respectively. Although the increase of peak current with increasing step potential, amplitude and square wave frequency are implicit, the parameters were optimized by making a compromise between the peak current increment and the shape of the peaks (Figure 13(A−C)).

Hence, step potential: 6 mV, amplitude: 35 mV and frequency: 20 Hz were taken as the optimum square wave parameters for further experiments.

### Calibration curve and limit of detection

**Figure 14:**
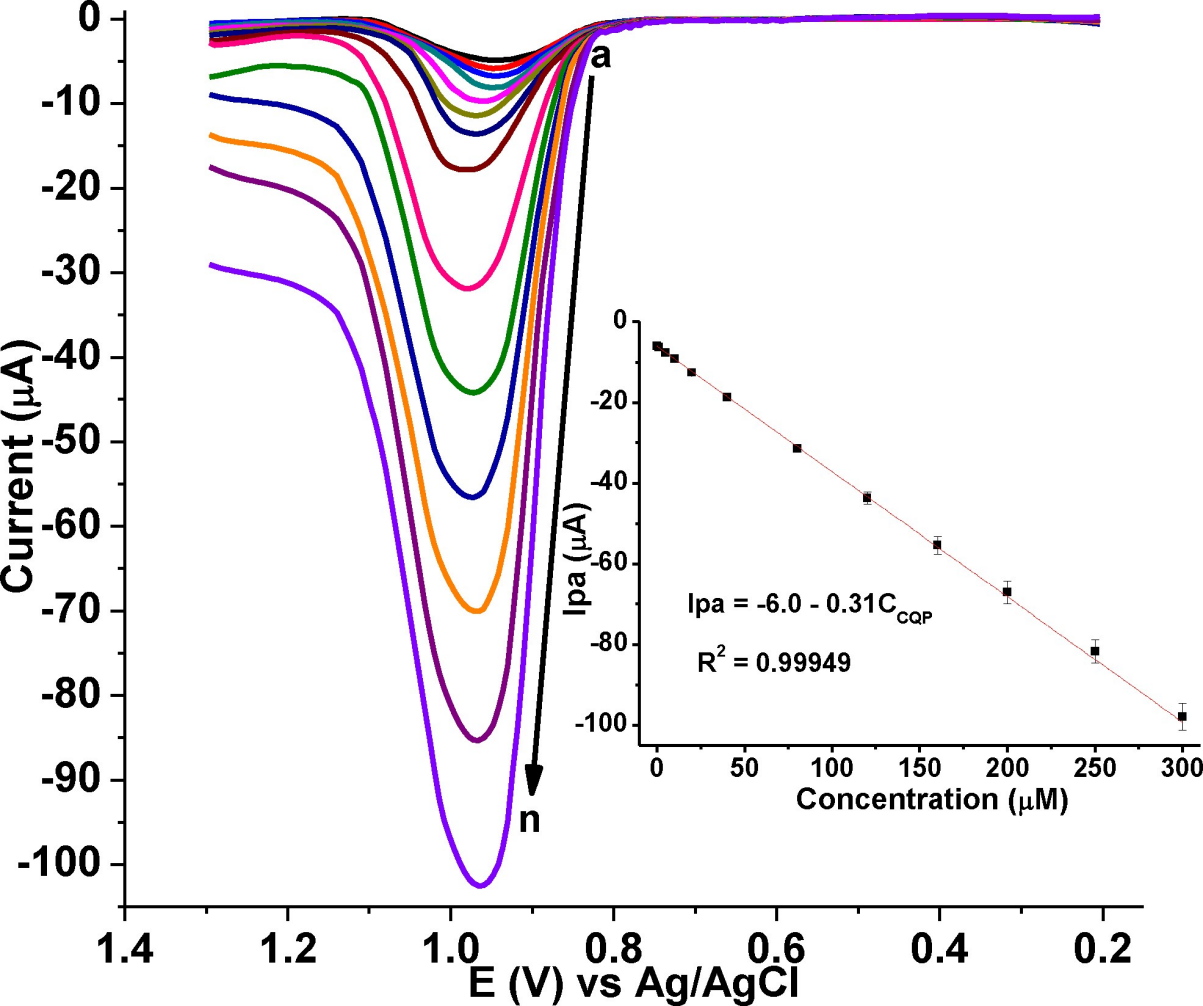
Blank subtracted AdSSWVs of various concentrations of CQP (a‐n: 0.005, 0.01, 0.1, 1.0, 5.0, 10.0, 20.0, 40.0, 80.0, 120.0, 160.0, 200.0, 250.0, and 300.0 μm, respectively) in pH 7.5 PBS at poly(DHRPCo)/GCE. Inset: plot of I_pa_ vs. concentration, with E_acc_=0.7 V; t_acc_=20 s; potential step=6 mV; amplitude=35 mV; and frequency=20 Hz.

In order to assess the applicability of the developed sensor (poly(DHRPCo)/GCE) for the determination of CQP in tablet formulations, human blood serum, and urine samples. The calibration curve was constructed in the CQP concentration range of 0.005–300.0 μM under optimized SWV parameters and solution pH (Figure 14) with %RSD below 4.1. The limit of detections of the method (LoD=3 s/m, *n=*7) and limit of quantification (LoQ=10s/m) were 0.39 and 1.36 nm, respectively.

### Analytical application of poly(DHRPCo)/GCE in real samples

#### Tablet samples

**Figure 15:**
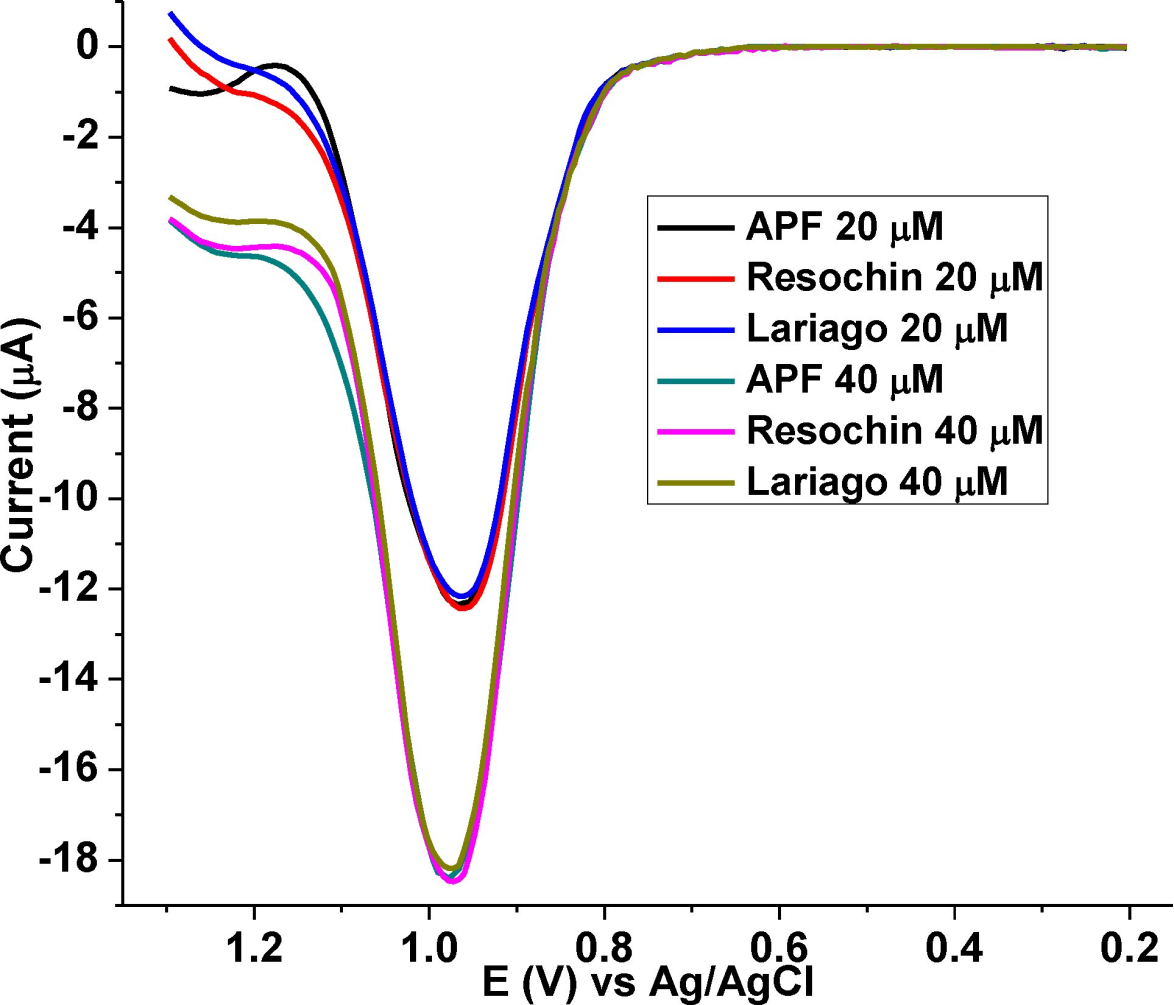
Blank corrected AdSSWVs of nominal (A) 20.0 and (B) 40.0 μm of CQP in pH 7.5 PBS in different tablet brands (APF, Resochin, and Lariago) at poly(DHRPCo)/GCE.

Under the optimum conditions (adsorption and SWV parameters and solution pH), the applicability of the developed sensor for determination of CQP amount in each tablet brand samples listed under section 2.5 were investigated. The detected levels of each brand were compared with the labeled CQP amount in the samples. The AdSSWVs for 20.0 and 40.0 μm of CQP for each tablet brand are presented in Figure 15, and the actual amount of CQP detected in each sample are summarized in Table 2.

**Table 2:** Summary of detected CQP and percent detected compared to the nominal content of each tablet brands.

CQP brand	Nominal CQP [μm]	Detected CQP [μM]^[a]^	Detected CQP [%]
APF	20.0	20.32±0.031	101.6
	40.0	39.70±0.029	99.25
Resochin	20.0	20.64±0.03	103.2
	40.0	40.00±0.033	100.0
Lariago	20.0	19.83±0.029	99.15
	40.0	39.35±0.027	98.40

[a] Detected mean CQP±RSD.

The analysis content in the range between 98.40–103.2 %, which is in excellent agreement with the labeled content of the tablets with RSD 3.3 %. Therefore, the developed method could be successfully applied for the determination of CQP in pharmaceutical formulations.

#### Human blood serum sample

**Figure 16:**
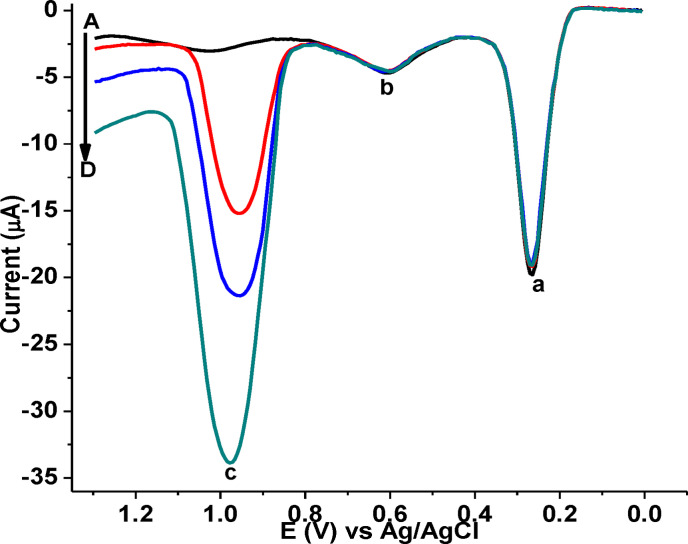
Background corrected AdSSWVs of poly(DHRPCo)/GCE in pH 7.5 PBS containing (A−D; unspiked human blood serum sample, A+20.0 μm, A+40.0 μm, and A+80.0 μm CQP, respectively, (peak a & b: unassigned, and c: CQP).

A small peak exist at the characteristic potential of CQP (curve A of Figure 16) indicates presence of CQP in the analyzed human blood serum sample while the detected amount very small below LoQ, appearance of a peak (Figure 16a&b) at a potential away from that of CQP indicates presence of a non‐assignable electroactive substance in the blood serum sample.

#### Human urine sample

**Figure 17:**
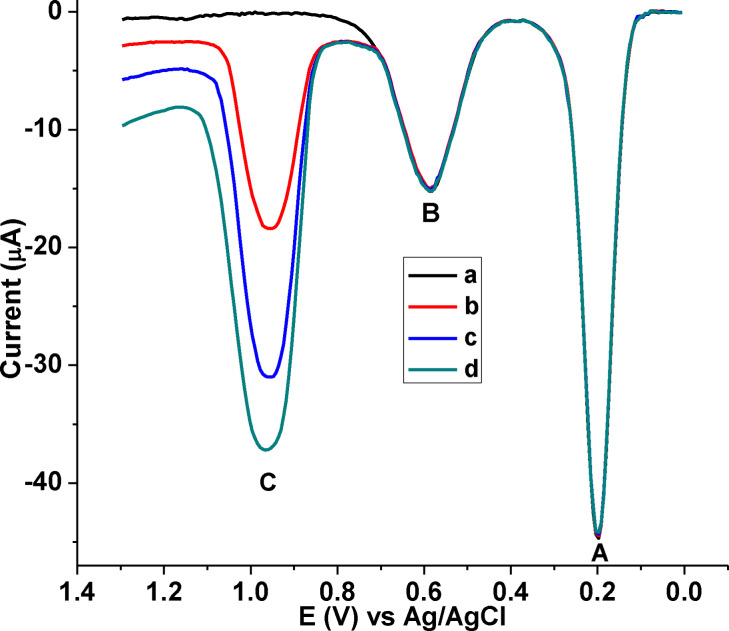
Blank subtracted AdSSWVs of poly(DHRPCo)/GCE in pH 7.5 PBS containing (a) unspiked urine sample, (b) a+40.0 μm CQP, (c) a+80.0 μm CQP, and (d) a+100.0 μm CQP, while (peak A, B & C represents UA, creatinine, and CQP, respectively) .

Absence of an observable peak at the characteristic potential of CQP (curve a of Figure 17) confirmed the absence of CQP in the human urine sample, the reliability of which was examined by spike recovery analysis. Among the two peaks that appeared at potentials away from the potential of CQP, peak‐A was assigned to uric acid, and the peak at about 690 mV (peak B) was assigned to creatinine.[^18^, ^22^]

### Validation of the developed method

#### Spiked recovery analysis

##### Human blood serum sample

As can be seen from Figure 16, a very small peak current for CQP observed (below LoQ) in the unspiked blood serum sample (peak c of curve A), confirmed by spiked standard CQP with increasing current in response to the spiked amount of CQP increase (peak c of curve B−D). In contrary the current for the peak (peak a & b) detected in the unspiked serum remained constant even with increasing amount of spiked CQP indicating that the peak was not for CQP. As shown in Table 3, spike recovery results in the range 99.35–100.28 % with %RSD under 3.3 %, validated the proposed sensor for determination of CQP in human blood serum samples.

**Table 3:** Recovery of 20.0, 40.0, and 80.0 μm CQP in spiked human blood serum.

Sample	CQP before spike (μM)	Added CQP (μM)	Found CQP (μM)^[a]^	% recovery
B	ND	20.0	19.87±0.032	99.35
C	ND	40.0	39.77±0.033	99.43
D	ND	80.0	80.22±0.031	100.28

ND not detected, [a] Detected mean CQP±RSD

##### Human urine sample

Spiked recovery of CQP in human urine sample was carried out by spiking the human urine sample with 40.0, 80.0 and 120.0 μM CQP (Figure 17). The AdSSWV of urine samples revealed a peak for uric acid, and creatinine, both with exactly constant current intensity regardless of level of spiked CQP. Besides, appearance of a peak (peak C) on the characteristic potential of CQP whose peak current increases with spiked CQP increases (curves b & c) confirmed absence of CQP in the unspiked human urine sample. Spike recovery result in a range of 99.03 %–100.32 % of CQP in human urine (Table 4) with %RSD under 3.7 % validated applicability of the developed method for determination of CQP in human urine.

**Table 4:** Spike recovery results of CQP in human urine samples.

Sample	CQP before spike [μm]	Added CQP [μm]	Found CQP [μm]^[a]^	% recovery
b	ND^[b]^	40.0	39.61±0.037	99.03
c	ND	80.0	80.00±0.024	100.00
d	ND	100.0	100.32±0.031	100.32

[a] Detected mean CQP±RSD; [b] ND: not detected,

##### Tablet samples

**Figure 18:**
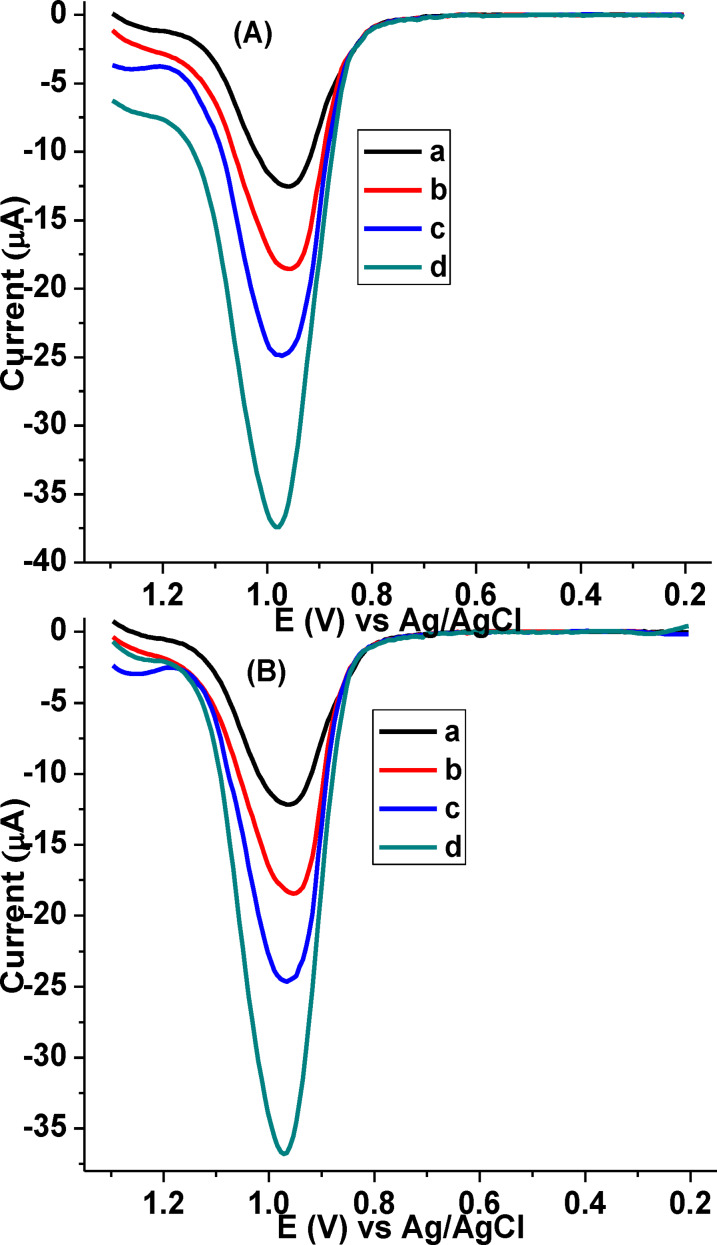
Background subtracted AdSSWVs of Resochin (A), and Lariago (B) brand tablet samples spiked with various concentrations of standard CQP (a‐d: 0.0, 20.0, 40.0, and 80.0 μm, respectively), at poly(DHRPCo)/GCE in PBS of pH 7.5.

To evaluate the validity of the developed method for the determination of CQP in tablet formulation samples, recovery studies was conducted at poly(DHRPCo)/GCE. The experiment was performed by spiking 20.0, 40.0 and 80.0 μm of CQP standard solution into 20.0 μm of Resochin, and Lariago brand tablet samples (Figure 18A and B). As shown in the Table 5, excellent recovery results in the range of 98.40–100.41 % were obtained, indicating that the proposed sensor can be effectively applicable for the determination of CQP in pharmaceutical samples.

**Table 5:** Summary of percentage recoveries of spiked standard CQP in Resochin, and Lariago brand pharmaceutical samples.

Tablet Sample	Spiked CQP [μM]	Expected CQP [μM]	Detected CQP [μM]^[a]^	Recovery [%]
Resochin	–	20.64	20.64±0.03	–
	20.0	40.64	40.32±0.035	98.40
	40.0	60.64	60.64±0.025	100.00
	80.0	100.64	100.97±0.030	100.41
Lariago	–	19.83	19.83±0.029	–
	20.0	39.83	39.68±0.031	99.25
	40.0	59.83	59.74±0.027	99.78
	80.0	99.83	99.03±0.032	99.0

[a] Detected mean CQP±RSD

### Interference study

**Figure 19:**
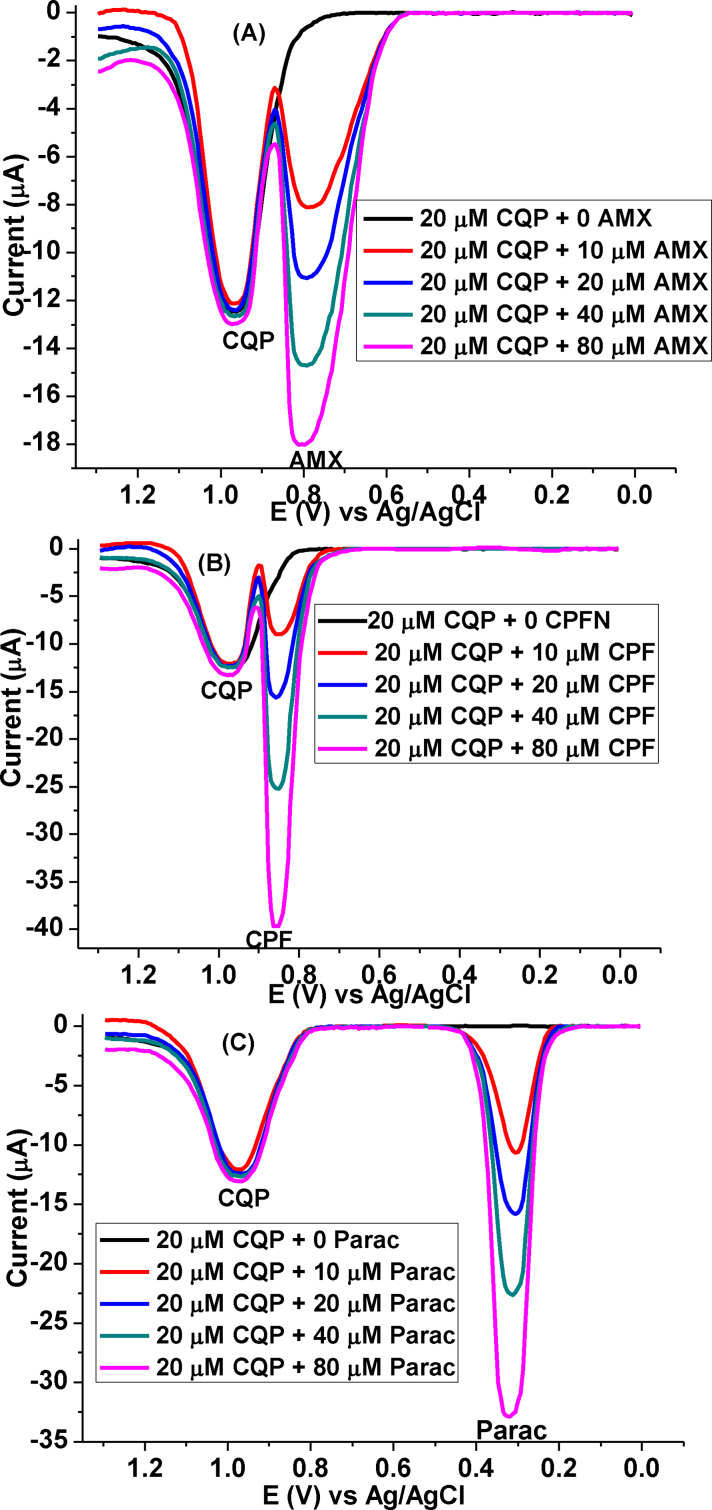
Blank corrected AdSSWVs of pH 7.5 PBS containing 20.0 μm CQP Resochin brand tablet solution at poly(DHRPCo)/GCE in the presence of (A) AMX, (B) CPF, and (C) Parac of various concentrations(0, 10.0, 20.0, 40.0, & 80.0 μm).

To further elaborate the applicability of the proposed method, the selectivity of the poly(DHRPCo)/GCE for the determination of CQP was evaluated in the presence of interferents amoxicillin (AMX), ciprofloxacillin (CPF), and paracetamol (Parac). The effect of each selected interferent was investigated at various concentrations of the interferents (Figure 19A−C) added to 20.0 μm Resochin brand CQP tablet sample. As shown in Table 6, the detected level of CQP in the presence of AMX, and Parac was the same as expected with an error of less than 4.6 %, which was observed additional peak as a result of the oxidation of AMX, CPF, and Parac. However, the peak current of CQP interfere (6.45) in 80.0 μm CPF, indicating the selectivity of the developed method (poly(DHRPCo)/GCE) for determination of CQP in real samples with the presence of potential interferents.

**Table 6:** Summary of interference study results of CQP in the presence of AMX, CPF, and Parac.

Interferent	Interferent added [μm]	Current response [μA]	Expected current [μA]	%error
AMX	0	12.4	12.4	–
	10.0	12.1	12.4	2.48
	20.0	12.3	12.4	0.81
	40.0	12.6	12.4	1.61
	80.0	12.9	12.4	4.03
CPF	0	12.4	12.4	—
	10.0	12.04	12.4	3.33
	20.0	12.3	12.4	0.81
	40.0	12.4	12.4	0.00
	80.0	13.2	12.4	6.45
Parac	0	12.4	12.4	—
	10.0	12.07	12.4	2.73
	20.0	12.37	12.4	0.24
	40.0	12.64	12.4	1.93
	80.0	12.97	12.4	4.60

### Stability and reproducibility studies

**Figure 20:**
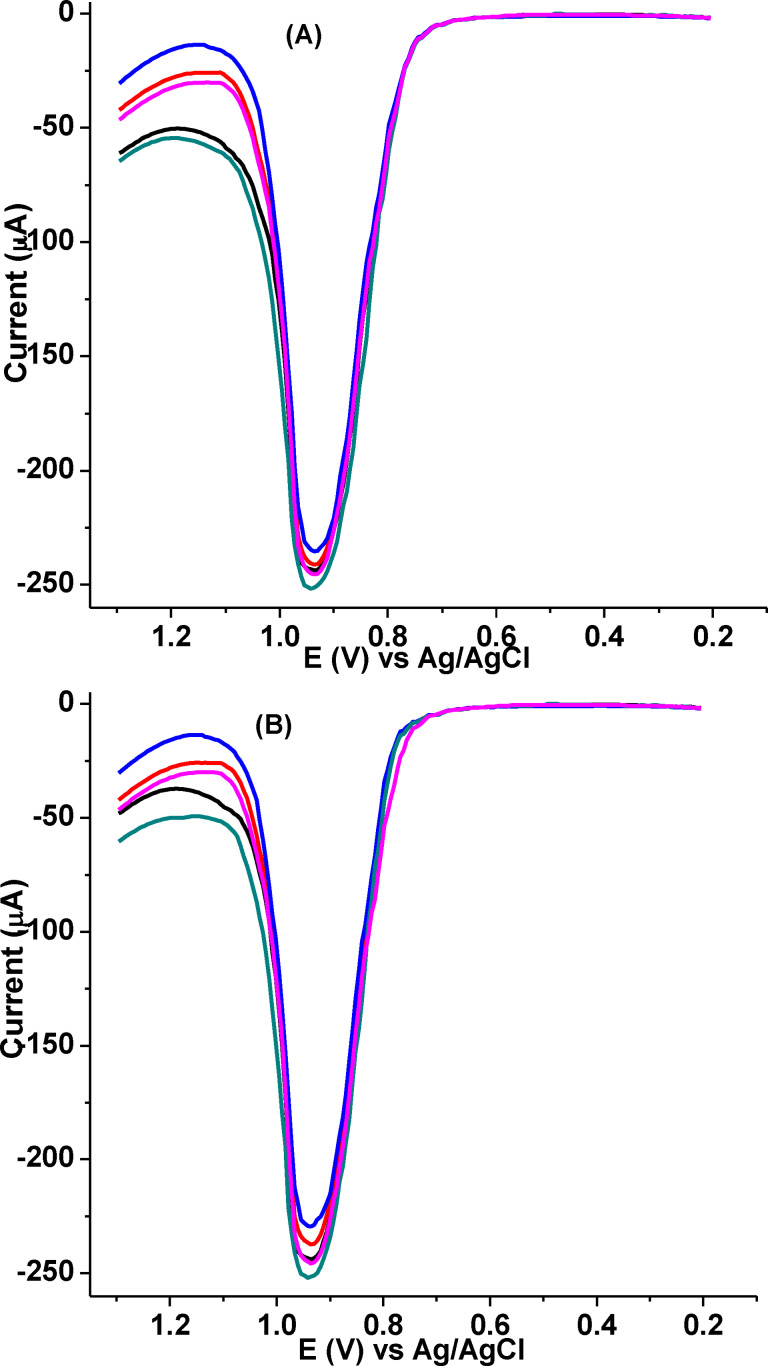
AdSSWVs for five repetitive measurements containing 1.0 mm CQP in pH 7.0 PBS at the surface of poly(DHRPCo)/GCE recorded in (A) 5 weeks interval of one week and (B) five different electrodes with E_acc_=0.7 V; t_acc_=20 s; potential step=6 mV; amplitude=35 mV; and frequency=20 Hz.

The long‐term (Inter‐day) stability of poly(DHRPCo)/GCE was examined by five successive AdSSWV measurements recorded at an interval of 1 week for 5 weeks with an error of only 2.50 % (%RSD) (Figure 20A). Similarly, the reproducibility of the developed sensor was evaluated by measuring the response of 1.0 mm CQP in pH 7.5 of PBS for five separate electrodes prepared using similar fabrication process, very good reproducibility result was obtained which showed the %RSD below 3.46 % (Figure 20B). Hence, it can be concluded that the stability and reproducibility of the proposed sensor, poly(DHRPCo)/GCE are adequate for CQP determination.

### Comparison of the present method with previously reported methods

The performance of the present sensor was compared with previous reports for the determination of CQP in terms of modifier used, linear dynamic range, and LoD (Table 7). Along with the lowest LoD and wide linear concentration range of the present method that of the previous reports, the developed method exhibits excellent results with the other investigations by using a simple modifying procedure, non‐toxic and cost effective modifier. Therefore, the present method using the synthesized complex modifier can be an excellent candidate for monitoring level of CQP in biological fluids and pharmaceutical formulation samples.

**Table 7:** Comparison of different electrochemical sensors reported for CQP determination.

Substrate	Modifier	Dynamic range [μm]	LoD [μm]	Ref.
CPE	dsDNA	0.1–10.0	0.03	[6]
CPE	MWCNTs	0.057–100.0	0.006	[9]
GCE	BDD	0.01–0.25	0.002	[17]
GCE	rGO@WS_2_	0.5–82.0	0.04–0.12	[20]
CPE	Cu(OH)_2_ nano‐wire	0.068–6.88	0.01	[31]
GCE	poly(DHRPCo)	0.005–300.0	0.00039	This work

## Conclusions

The present study established a simple, nontoxic, cost‐effective, highly sensitive and selective method for the fabrication of voltammetric sensors for CQP based on the potentiodynamic electropolymerization of diresorcinate‐1,10‐phenanthrolinecobalt(II) complex on GCE. The fabricated sensor showed significant peaks current enhancement and over potential reduction as compared to the bare GCE. Poly(DHRPCo)/GCE was successfully utilized for AdSSWV determination of CQP with a promising wide linear dynamic range and lowest limit of detection. The sensor was successfully applied for determining CQP in human blood serum, urine, and tablet formulations of three brands with excellent recovery values. The effect of potential interferents such as AMX, CPF and Parac on the determination of CQP using the proposed sensor was also examined. Besides the simplicity of electrode modification process, stability and repeatability, excellent recovery results for pharmaceutical analysis suggest the potential of poly(DHRPCo)/GCE for direct determination of CQP in various real samples.

## Conflict of interest

The authors declare no conflict of interest.

1

## Data Availability

The data that support the findings of this study are available from the corresponding author upon reasonable request.
